# Temporal response patterns of human gut microbiota to dietary fiber

**DOI:** 10.1002/imt2.70046

**Published:** 2025-05-10

**Authors:** Xiaotong Lin, Chaoxun Wang, Biao Liu, Yin Zhu, Rui Zhai, Chenhong Zhang

**Affiliations:** ^1^ State Key Laboratory of Microbial Metabolism School of Life Sciences and Biotechnology, Shanghai Jiao Tong University Shanghai China; ^2^ Department of Endocrinology Shanghai Pudong Hospital, Fudan University Pudong Medical Center Shanghai China; ^3^ Adfontes (Shanghai) Co., Ltd Shanghai China; ^4^ Shanghai Jiao Tong University Sichuan Research Institute Chengdu China

**Keywords:** dietary fiber, gut microbiota, overweight, time series, type 2 diabetes mellitus

## Abstract

The gut microbiota is a highly dynamic and complex ecosystem. However, the processes by which its members respond to dietary fibers remain incompletely understood. Here, we performed daily sampling over a 14‐day observational period under the habitual diet, followed by a 14‐day dietary fiber intervention in overweight participants with and without type 2 diabetes mellitus. By combining daily sampling, guild‐level approach, and time‐series analysis, we revealed diverse temporal response patterns among various microbiota members that are often missed by conventional sampling. These patterns were closely linked to their genetic capacities for carbohydrate utilization and transport. Moreover, time‐delayed analysis of longitudinal multi‐omics data identified specific metabolites that potentially mediate the beneficial effects of gut microbiota on host metabolism. Overall, our findings demonstrate the necessity of high‐frequency sampling for capturing dynamic microbial responses and offer reliable targets for mechanistic investigations.

## Highlights

- High‐frequency sampling revealed dynamic gut microbiota responses to dietary fiber that were missed by pre‐to‐post comparisons, linking these patterns to microbial genetic capacities for carbohydrate metabolism.
- Guild‐based analyses of individual‐level networks uncovered stable interactions among co‐occurring microbial members.
- Time‐delayed analyses identified microbiota–metabolite associations with positive implications for host health, suggesting potential targets for causal validation.

## Graphical abstract

In this study, 19 overweight participants, with or without type 2 diabetes mellitus, underwent 14 days of normal‐diet observation followed by 14 days of dietary fiber intervention. Fecal samples and continuous glucose monitoring data were collected daily throughout the 28‐day period. Using guild‐based analysis of individual‐level networks and time‐series approaches, we captured the dynamic responses of gut microbiota, revealing multiple abundance‐shift patterns that are often overlooked by conventional sampling strategies. By integrating multi‐omics data with time‐delayed analysis, we further identified key microbial members and metabolites associated with host metabolic improvements, thereby providing reliable targets for subsequent mechanistic investigations.

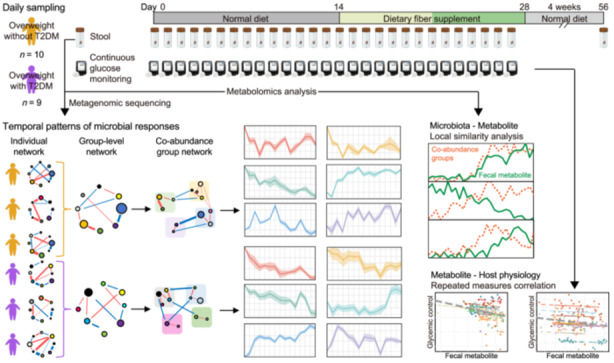

## INTRODUCTION

Dietary fiber interventions targeting gut microbiota are widely used to improve human health [1]. These beneficial effects are achieved through complex cross‐feeding interactions among various gut microbes [2]. The cross‐feeding process involves primary degraders that hydrolyze complex polysaccharides into mono‐ or oligosaccharides, which are then utilized and fermented by members of subsequent trophic levels to produce short‐chain fatty acids (SCFAs) [2]. Identifying the key members that utilize dietary fiber and their dynamic responses over time is a critical research priority. Previous work has made progress in this area. For instance, Liu et al. identified primary degraders of inulin by applying an ecological model to time‐series data [3]. Similarly, Wei et al. tracked the inulin‐induced enrichment of *Parabacteroides distasonis* through ^13^C labeling in a mouse model [4]. Furthermore, Creswell et al. developed a tailored computational tool to analyze longitudinal data and identify microbial groups exhibiting similar kinetic responses during glycans intervention in healthy participants [5]. However, the variations in findings across different studies and a lack of mechanistic validation have limited our understanding of gut microbiota dynamics.

Currently, the majority of clinical studies of dietary interventions rely on cross‐sectional study design or pre‐to‐post intervention comparisons with limited sampling points. This conventional sampling approach fails to capture the complete variation of microbiota during the trial [6], as microbiota rapidly respond to environmental changes, with shifts occurring within 24 h of dietary modifications [7, 8]. Moreover, microbial changes exhibit cumulative effects over time as evidenced by genetic adaptations accumulating across generations [9]. For instance, in *Bacteroides thetaiotaomicron*, adaptive mutations induced by standard and Western diets can accumulate in as little as 2 weeks [10]. Theoretically, host physiological responses reflect the cumulative effects of dynamic microbial changes throughout the intervention period, rather than solely the microbiota composition at the endpoint. These basic biological principles suggest that high‐frequency sampling is necessary for the investigation of microbiota dynamics. Additionally, integration of high‐resolution multi‐omics data is crucial for identifying key microbial mediators, providing a foundation for revealing in‐depth mechanisms or causal relationships. This sampling approach has been proven effective in environmental microbiome studies, as demonstrated by Seitz et al., who employed daily sampling to elucidate how cover crop root exudates modulate microbial nitrogen cycling and phytohormone biosynthesis in soil microbiomes [11].

Understanding the dynamic microbiome also requires recognition of its fundamental nature as a complex adaptive system [12]. In this ecosystem, microbial members form interactions and self‐organize into functional units known as guilds or co‐abundance groups (CAGs), which collectively influence host metabolism [13]. Members within the same guild consistently respond to environmental changes, particularly nutritional shifts, by growing or declining together [14]. Notably, microbiota members performing essential biological functions exhibit stable interactions, demonstrating consistent positive or negative associations over time and across diverse physiological conditions [15]. Furthermore, while the inherent high dimensionality and sparsity of microbiome datasets pose significant challenges for applying various statistical models, guild‐based analysis provides an alternative approach to address these limitations. For instance, Wu et al. demonstrated the effective reduction of approximately 2 million genes to 161 genomes and subsequently to 18 guilds, enabling the identification of guild‐disease phenotype associations and the construction of robust predictive models [13]. Thus, guild‐based analysis aligns with the ecological nature of gut microbiota while providing a powerful dimensionality reduction approach, making it essential for exploring microbiome‐host relationships.

In this study, we selected dietary fiber as an effective perturbation based on substantial evidence demonstrating its modulatory effects on microbiota and host health. We conducted a longitudinal trial to precisely characterize the dynamic responses of the microbiota to dietary fiber in 19 overweight participants, both with and without type 2 diabetes mellitus (T2DM). Daily fecal samples were collected to monitor changes in microbiota composition and metabolites. Concurrently, host glycemic variability was tracked using continuous glucose monitoring (CGM). Our analytical framework integrated daily sampling, time‐series analysis, and CAG analysis to examine the dynamic responses of gut microbiota to dietary fiber with enhanced temporal resolution. Functional gene traits associated with carbohydrate utilization and transport capabilities in each cluster were subsequently characterized. Furthermore, by incorporating time‐delayed analysis within our multi‐omics data integration, we identified two novel potential interactions among microbiota, metabolites, and host metabolism. Overall, our study enhances our understanding of the temporal dynamics of gut microbiota, informing reliable targets for mechanistic investigations.

## RESULTS

### Dietary fiber induced rapid glycemic control improvements and microbiota shifts

**FIGURE 1:**
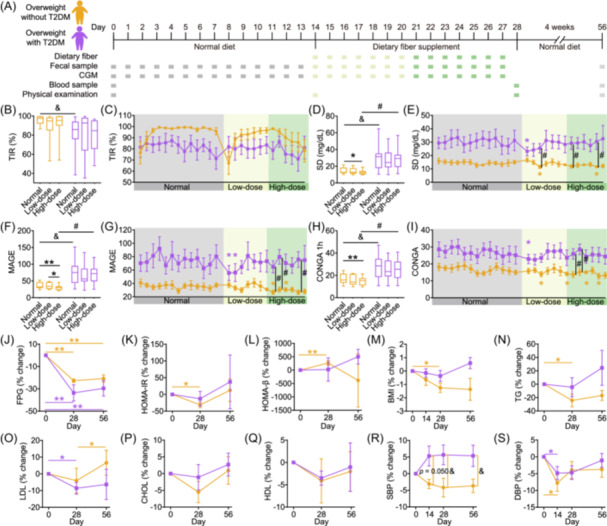
Dietary fiber rapidly enhanced participants' glucose stability. (A) Study design and measurements. The study consisted of a 2‐week normal diet, followed by a 2‐week dietary fiber intervention, and a 4‐week follow‐up period. The normal diet phase (normal) is represented in gray, with the low‐dose dietary fiber intervention phase (low‐dose) in light green and the high‐dose dietary fiber intervention phase (high‐dose) in green. The period 4 weeks post‐intervention (follow‐up, FU, also referred to as Day 56) appears in light gray. Changes in percent time in range (between 70 and 180 mg/dL, B and C), standard deviation of the daily glucose (D, E), mean amplitude of glycemic excursion (F, G), continuous overlapping net glycemic action (H, I) during the trial. Boxes show the medians and interquartile range (IQR), with the whiskers extending to the lowest and highest values. In the line chart, data are presented as mean ± standard error of the mean (SEM). Across groups, Mann–Whitney *U* test (two‐tailed) was utilized during the normal diet phase (^&^indicates *p* < 0.05), while one‐way analysis of covariance (ANCOVA) was conducted throughout the dietary fiber intervention phase, adjusted by the mean values of the normal diet phase (^#^indicates *p* < 0.05). Within each group, Wilcoxon matched‐pairs signed‐rank test (one‐tailed) was performed to evaluate the differences at various intervention time points compared to the mean values of the normal diet phase (*indicates *p* < 0.05 and **indicates *p* < 0.01). Changes in FPG (J), HOMA‐IR (K), HOMA‐β (L), BMI (M), TG (N), LDL (O), CHOL (P), HDL (Q), SBP (R), and DBP (S) for subjects during the trial. Data are presented as percentage changes from Day 0 (mean ± SEM). Mann–Whitney *U* test (two‐tailed) was used for intergroup comparisons at the same time point (^&^indicates *p* < 0.05), and the Wilcoxon matched‐pairs signed‐rank test (two‐tailed) was utilized for intra‐group comparisons over different time points with Day 0 (*indicates *p* < 0.05 and **indicates *p* < 0.01). FPG: fasting plasma glucose, HOMA‐IR: homeostasis model assessment of insulin resistance, HOMA‐β: homeostasis model assessment of beta‐cell function, BMI: body mass index, TG: triglycerides, LDL: low‐density lipoprotein, CHOL: total cholesterol, HDL: high‐density lipoprotein, SBP: systolic blood pressure, DBP: diastolic blood pressure.

Between August and October 2021, we recruited 20 overweight individuals, 10 of whom had T2DM. At baseline, body mass index (BMI) was comparable between groups (Overweight group: 27.78 ± 1.12 kg/m^2^, T2D group: 27.75 ± 0.80 kg/m^2^). Initial phenotypic characteristics were also similar between groups, except for fasting plasma glucose (FPG) and HbA1c levels (Table S1). Our study comprised a 2‐week observation phase with participants following their usual diet (Days 0–13, Figure 1A), a 2‐week dietary fiber supplementation phase (Days 14–27, Table S2), and a 4‐week follow‐up (Days 28–56). During the supplementation phase, participants consumed 18 g/day from Day 14 to Day 20 (low‐dose phase) and 36 g/day from Day 21 to Day 27 (high‐dose phase). Patients with T2DM continued their antidiabetic medications throughout the trial (Table S3). All 10 participants in the Overweight group and nine in the T2D group completed the study (Figure S1A). Daily fecal samples were collected for microbiota composition and metabolite analyses, while glycemic excursions were monitored using continuous glucose monitoring (CGM, Table S4).

Time in range (TIR), the percentage of time per day with glucose levels between 70 and 180 mg/dL, served as our primary outcome. During the fiber intervention, TIR remained above 70% in both Overweight and T2D groups, aligning with effective T2DM management [16], despite no significant changes compared to the normal diet phase (Figure 1B,C). Secondary outcomes included glycemic variability (GV), blood lipid profile, blood pressure measurements, and BMI. GV was assessed using standard deviation (SD), mean amplitude of glycemic excursions (MAGE), and continuous overlapping net glycemic action (CONGA). Compared with the normal diet phase, GV decreased during the fiber intervention in both groups (Figure 1D–I). Daily data analysis revealed significant GV reductions following the initiation of supplementation (Figure 1E,G,I), confirmed by change‐point analysis of individual time series data (Figure S1B–G). Dietary fiber supplements also significantly reduced FPG from baseline (Day 0) to the end of the intervention (Day 28) and follow‐up (Day 56) in both groups (Figure 1J). The Overweight group showed more pronounced improvements in HOMA‐IR, HOMA‐β, BMI, and triglycerides (Figure 1K–N), while low‐density lipoprotein (LDL) showed a greater decrease in the T2D group (Figure 1O). Other secondary outcomes remained unchanged (Figure 1P–S).

**FIGURE 2:**
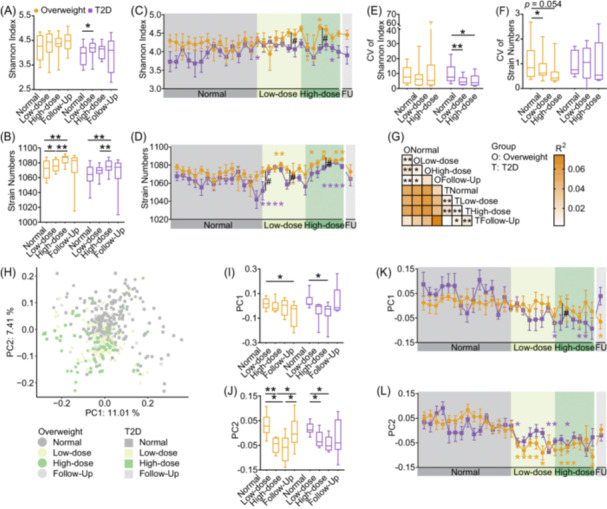
Dietary fiber rapidly increased participants' gut microbiota α‐diversity and altered structure. (A–F) Changes in diversity (Shannon index) and richness (number of strains) of the gut microbiota. (A) Shannon index across different phases of the trial. (B) Number of strains across different phases of the trial. (C) Daily changes in the Shannon index. (D) Daily changes in number of strains. (E) Coefficient of variation of Shannon index. (F) Coefficient of variation of strain numbers. (G) The subject‐stratified PERMANOVA test based on Bray–Curtis dissimilarity with 999 permutations, Benjamini–Hochberg (BH) adjusted *p* < 0.05. (H) Covariate‐adjusted principal coordinates analysis (aPCoA) of Bray‐Curtis dissimilarity at the high‐quality metagenome‐assembled genomes (HQMAG) level, visualizing microbiota structural diversity with dots representing samples. (I–L) Changes in gut microbiota on PC1 and PC2. (I) PC1 across different phases of the trial. (J) PC2 across different phases of the trial. (K) Daily changes in PC1. (L) Daily changes in PC2. Boxes show the medians and IQRs, with whiskers illustrating the lowest and highest values. In the line chart, data are presented as mean ± SEM. Across groups, Mann–Whitney *U* test (two‐tailed) was used for intergroup comparisons during the normal diet, while one‐way ANCOVA was used throughout the dietary fiber intervention phase, adjusted by the mean values of the normal diet phase (^#^indicates *p* < 0.05). Within each group, Wilcoxon matched‐pairs signed‐rank test (two‐tailed) was performed to evaluate the differences at various intervention time points compared to the mean values of the normal diet phase (*indicates *p* < 0.05 and **indicates *p* < 0.01).

To explore the changes in gut microbiota, we performed shotgun metagenomic sequencing on 433 fecal samples and de novo assembled 1100 high‐quality metagenome‐assembled genomes (HQMAGs, Tables S5, 6). Compared with the normal diet phase, dietary fiber increased microbial diversity (Shannon index) and richness (strain numbers) in both groups (Figure 2A,B), with significant changes occurring within the first 3 days of intervention (Figure 2C,D), confirmed by change‐point analysis in most participants (Figure S2). Dietary fiber also enhanced the stability of alpha diversity, as shown by reduced coefficients of variation in Shannon index for the T2D group and in strain numbers for the Overweight group (Figure 2E,F).

Individual variability explained most of the observed variation in microbiota composition (75.03%, Figure S3A,B), consistent with previous studies [17]. After adjusting for participant variability, we found that dietary fiber induced significant microbiota shifts from the normal diet phase to low‐dose and high‐dose phases in both groups (Figure 2G–L). Daily sampling revealed that rapid changes occurred as early as the second day of the intervention (Figure 2L), as confirmed by Bray–Curtis distance change‐point analysis for most participants in both groups (Figure S3C,D). Overall, dietary fiber effectively improved participants' glucose metabolism while rapidly enhancing gut microbiota diversity and altering microbial structure in both groups, with these changes occurring at the onset of the intervention.

### Dynamics of microbial responses to dietary fiber

**FIGURE 3:**
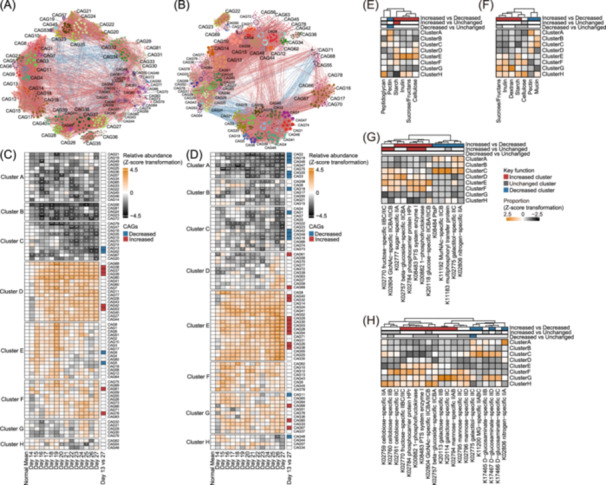
CAGs exhibited various temporal response patterns to dietary fiber. Co‐abundance network in the Overweight group (A) and the T2D group (B) during the trial, denoted as GOverweight (1000, 17,876) and GT2D (902, 10,221), where the numbers in parentheses indicate the number of nodes and edges, respectively. Co‐abundance correlations between HQMAGs were calculated using FastSpar for each subject. Correlations were incorporated into each group's co‐abundance network, requiring consistently significant (*p* ≤ 0.05) positive or negative correlations across at least six participants within each group. Node color signifies its co‐abundance group (CAG) membership, and node size reflects the degree. Edges between nodes represent correlations, red and blue colors indicate positive and negative correlations, respectively. For clarity, only lines corresponding to correlations with magnitudes ≥ 0.7 in the Overweight group and ≥ 0.6 in the T2D group were drawn. The heatmaps show the temporal patterns of CAG abundance fluctuations during dietary fiber intervention in the Overweight group (C) and the T2D group (D), relative to the average values from the normal diet phase. Left: CAGs were clustered into distinct temporal patterns based on their abundance changes using dynamic time warping distance and Ward's D2 linkage. Middle: Significant variations in daily CAGs abundance throughout the intervention were assessed through linear mixed models. Significance levels are denoted by asterisks with ^+^indicating *p* < 0.1, *indicating *p* < 0.05, **indicating *p* < 0.01, ***indicating *p* < 0.001. Right: CAGs showing significant abundance shifts between the end of the intervention (Day 27) and the end of normal diet (Day 13). Significant increases and decreases in abundance are colored red and blue. The heatmaps show the average proportion of CAZy genes (E, F) and phosphotransferase system (PTS) KEGG orthologs (KOs, G and H) within clusters for the Overweight group (E, G) and the T2D group (F, H). For carbohydrate substrate utilization and transfer, functional genes and KOs were identified in each HQMAG. At the CAG level, the proportion of CAZy genes for a particular substrate was calculated as the number of CAZy genes involved in its utilization divided by the total number of CAZy genes predicted within each CAG. Similarly, the proportion of PTS‐associated KOs was determined by the number of such KOs divided by the total count of KOs predicted in each CAG. Clusters were categorized according to the significant post‐intervention changes in CAG abundance within each cluster, classified as increased, unchanged, and decreased clusters. Mann–Whitney *U* test (two‐tailed) was utilized to analyze the differences in CAZy gene and KOs proportions among the three cluster categories (*p* < 0.05). Colors highlight clusters with significantly higher proportions: red indicates increased clusters, gray indicates unchanged clusters, and blue indicates decreased clusters. MG: 2‐O‐α‐mannosyl‐d‐glycerate, GlcNAc: N‐acetylglucosamine, MurNAc: N‐Acetylmuramic acid.

We partitioned CAGs based on stable inter‐microbial relationships to identify microbial responses to dietary fiber at the guild‐level (Figure S4). After constructing individual co‐abundance networks (Figures S5, 6), we retained significant and consistent correlations in over 60% of participants in each group. In total, we selected 1000 HQMAGs in the Overweight group and 902 in the T2D group, accounting for 96.2 ± 0.25% and 94.46 ± 0.30% of total abundance in each group sample, respectively (Table S7). These HQMAGs were subsequently organized into 82 CAGs in the Overweight group and 78 in the T2D group (Figure 3A,B and Tables S8, 9).

Instead of using pre‐to‐post intervention comparison, we employed time‐series analysis to capture daily microbial changes, better reflecting the dynamic nature of gut microbiome [18]. Conventional‌ analysis detected shifts in 11 and 26 CAGs for the Overweight and T2D groups, respectively. However, linear mixed models (LMMs) based on our high‐frequency sampling data identified significant daily changes in a substantially larger number of CAGs (60 in the Overweight group and 69 in the T2D group) during the fiber intervention (Figure 3C,D). These findings highlighted the advantages of longitudinal data in capturing broader and more comprehensive microbial responses to dietary fiber.

Through clustering based on dynamic time‐warping distances, we identified eight temporal patterns of microbial responses in the Overweight group, with Clusters G and H maintaining stable abundances during the intervention (Figure 3C and Table S10). Clusters D, E, and F showed patterns of increased abundance, primarily composed of species known for utilizing carbohydrate polymers and producing SCFAs. Cluster D exhibited significant increases from the second day throughout the intervention. Three CAGs within this cluster were predominantly composed of *Bifidobacterium* species (*B. longum* and *B. pseudocatenulatum*), which can rapidly increase in the fiber‐rich environment through their unique “bifid shunt” pathway to efficiently convert carbohydrates into ATP [19]. Six other CAGs in Cluster D were dominated by SCFAs producers from Firmicutes, including *Agathobacter faecis* [20], *Blautia_A massiliensis* [21], *Fusicatenibacter saccharivorans* [22], *Faecalibacterium prausnitzii_G* [20], *F. prausnitzii*, and *Roseburia hominis* as dominant species. Cluster E increased notably during the low‐dose phase, followed by a decline in the high‐dose phase. Of its 16 CAGs, 12 were dominated by SCFA‐producing *Lachnospiraceae*, including *Anaerostipes hadrus* [20], *Mediterraneibacter faecis* [23], *Roseburia inulinivorans* [24], *Agathobacter faecis* [20], *Anaerobutyricum hallii* [25], *Anaerobutyricum soehngenii* [26, 27], and *Bariatricus comes* [20]. Additionally, four other CAGs in Cluster E were dominated by *Blautia_A wexlerae* and *Blautia_A faecis*, which produce acetic acid and lactate from carbohydrates, supporting other butyrate‐producing bacteria [28]. Cluster F exhibited an increasing trend throughout the intervention, with six out of 11 CAGs dominated by butyrate‐producing species from Firmicutes, including *Agathobacter rectalis* [29], *Agathobaculum butyriciproducens* [30], *F. prausnitzii_G*, *F. prausnitzii_C*, and *Clostridium_A leptum* [31]. Across Clusters D, E, and F, dominant species were mainly from Firmicutes and were known for SCFA production, reflecting their competitive advantage under fiber supplementation through enhanced glycan‐binding capacity [32]. Despite these similarities, the distinct temporal patterns among clusters likely resulted from species‐specific variations in carbohydrate metabolism enzymes and environmental adaptabilities.

Clusters A, B, and C in the Overweight group showed distinct decreasing patterns. Cluster A, dominated by pH‐sensitive *Bacteroides* species and potential pathogens including *Klebsiella pneumoniae* [33], *Parasutterella excrementihominis* [34], and *Enterocloster bolteae* [35], exhibited a major decline during the low‐dose phase, along with four CAGs characterized by high species diversity. Cluster B exhibited a sustained decline from the second day of the intervention, featuring acid‐sensitive *Bacteroides spp*. and *Phocaeicola vulgatus* [36]. Cluster C declined primarily during the high‐dose phase, with four of its CAGs dominated by species associated with various pathological conditions, including *Bilophila wadsworthia* [37], *Clostridium_AQ innocuum* [38], *Escherichia coli_D* [39], and *Haemophilus_D parainfluenzae* [40], and two CAGs showing high species diversity. These findings suggested that CAGs exhibiting abundance decline displayed characteristics of pH sensitivity, pathogenic potential, or taxonomic complexity.

Results from time‐series analysis differed from those of pre‐to‐post comparisons. While pre‐to‐post analysis identified seven increased CAGs, it missed the transient increase of Cluster E during the low‐dose phase, and misclassified CAGs 5 and 9 within Cluster E as decreased. Furthermore, this conventional approach detected only two decreased CAGs, overlooking diverse declining patterns. These findings highlighted the methodological advantages of daily sampling and time‐series analysis, enabling more comprehensive characterization of microbial response dynamics.

In the T2D group, we identified eight temporal patterns, many of which mirrored those observed in the Overweight group (Figure 3D and Table S11). Stable patterns were observed in Cluster H. Among increasing clusters, Cluster E in the T2D group and Cluster D in the Overweight group exhibited rapid and consistent growth, which contained SCFA producers such as *Bifidobacterium pseudocatenulatum*, *F. prausnitzii, F. prausnitzii_G, Fusicatenibacter saccharivorans*, and *Roseburia hominis*. Similarly, Cluster F in the T2D group and Cluster E in the Overweight group initially increased during the low‐dose phase, both containing *Anaerobutyricum soehngenii* and *Blautia_A wexlerae*. Moreover, both Cluster G in the T2D group and Cluster F in the Overweight group demonstrated a comparable upward trend but differed in dominant species. Regarding declining patterns, Cluster A in the T2D group exhibited rapid decreases, similar to Cluster B in the Overweight group, both of which contained pH‐sensitive *Phocaeicola vulgatus*. Additionally, Cluster D in the T2D group and Cluster A in the Overweight group initially decreased and later increased. In summary, parallel temporal patterns and phylogenetic traits observed across groups suggested that microbial responses to dietary fiber are similar across hosts.

Functional gene analysis further revealed the genetic capacities associated with temporal response patterns. In both groups, CAGs in the increased clusters exhibited higher proportions of CAZy genes involved in starch, inulin, and sucrose/fructans utilization (Figure 3E,F and Table S12). These clusters also showed greater proportions of KEGG orthologs (KOs) associated with the phosphotransferase system (PTS), including phosphocarrier protein, phosphoenolpyruvate‐protein phosphotransferase, and beta‐glucoside transport proteins (Figure 3G,H and Tables S13, 14). Conversely, declining CAGs had higher proportions of CAZy genes for pectin utilization and KOs related to galactitol and nitrogen transport (Table S15). These findings demonstrated that CAGs with similar abundance trends shared consistent functional traits in carbohydrate metabolism and transport, aligning with their taxonomic similarities.

### Temporal correlation of microbiota and fecal metabolites in glycemic regulation

**FIGURE 4:**
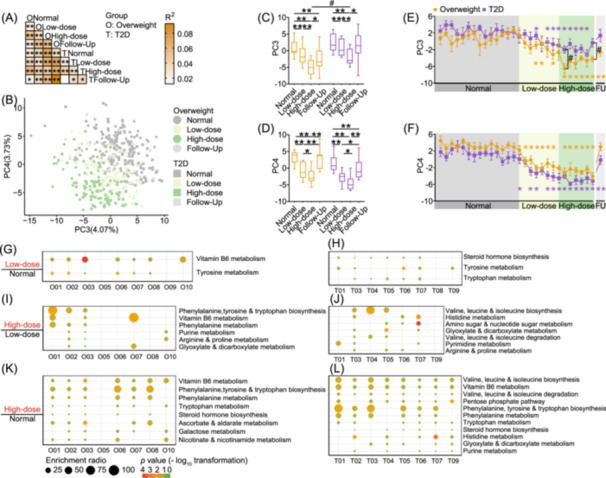
Dietary fiber rapidly altered participants' fecal metabolomics profiles. (A) PERMANOVA test (Euclidean dissimilarity) with 999 permutations, BH‐adjusted *p* < 0.05. (B) Principal component analysis (PCA) of fecal metabolite levels (Euclidean dissimilarity), with individual dots representing samples. (C–F) Changes in fecal metabolome on PC3 and PC4. (C) PC3 across different phases of the trial. (D) PC4 across different phases of the trial. (E) Daily changes in PC3. (F) Daily changes in PC4. Boxes show the medians and IQRs, whiskers extend to the lowest and highest values. In line charts, data are presented as mean ± SEM. Across groups, Mann–Whitney *U* test (two‐tailed) was used for intergroup comparisons during the normal diet phase, while one‐way ANCOVA was utilized throughout the dietary fiber intervention phase, adjusted by the mean values of the normal diet phase (^#^indicates *p* < 0.05). Within each group, Wilcoxon matched‐pairs signed‐rank test (two‐tailed) was performed to evaluate the differences at various intervention time points compared to the mean values of the normal diet phase (*indicates *p* < 0.05 and **indicates *p* < 0.01). The pathway enrichment analysis of fecal differential metabolites based on KEGG across various dietary phases are shown in the Overweight group (G, I, K) and the T2D group (H, J, L). (G, H) The enriched pathways in the low‐dose diet phase compared with the normal diet phase. (I, J) The enriched pathways during the high‐dose diet phase compared with the low‐dose diet phase. (K, L) The enriched pathways in the high‐dose diet phase compared with the normal diet phase.

Global untargeted metabolomics analysis revealed that fecal metabolome showed less individual variability than the microbiota composition, suggesting that microbial functions were similar across participants despite compositional variability (Figure S7). Both groups exhibited significant metabolome shifts during intervention phases (Figure 4A–D), with changes detectable as early as the first day of the low‐dose phase (Figure 4E,F). Pathway enrichment analysis of upregulated metabolites revealed enhanced amino acid metabolism during the low‐dose phase in the majority of participants from both groups (Figures 4G,H, S8). Notably, vitamin B6 metabolism was further upregulated in three nondiabetic overweight participants at higher doses (Figure 4I,J). High‐dose intervention led to a broader range of upregulated pathways, particularly those related to phenylalanine and tryptophan metabolism (Figure 4K,L). Overall, these results demonstrated that dietary fiber rapidly and substantially induced changes in participants' fecal metabolome.

**FIGURE 5:**
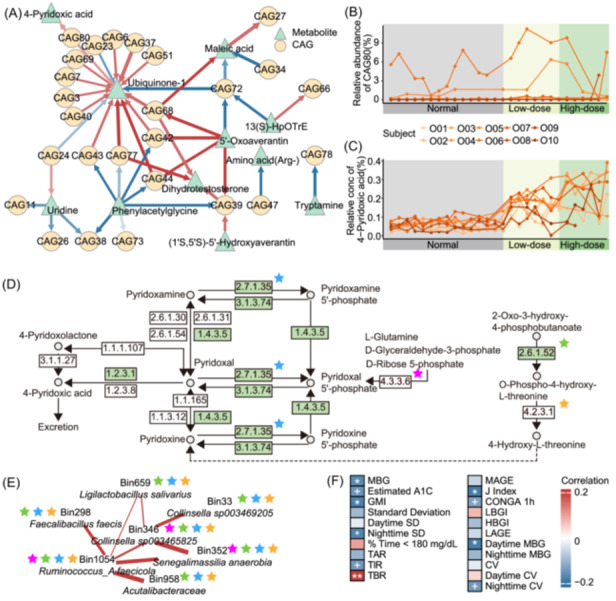
Role of 4‐Pyridoxic acid in the association between gut microbiota and host glycemic stability in the Overweight group. (A) Significant time‐delayed correlations between CAGs and fecal metabolites. Arrows in local similarity analysis represent the direction of time‐delayed relationships, with red indicating positive correlations and blue indicating negative correlations. The thickness of the arrow line represents the absolute value of the local similarity score, while the color intensity represents the duration of the time delay between nodes. The darker the arrow, the shorter the time delay. Changes in CAG80 (B) and 4‐Pyridoxic acid (C) in the Overweight group during the trial. Data are presented as mean ± SEM. (D) The KOs in vitamin B6 metabolism (KEGG pathway #00750) predicted in Overweight group's CAG80 are highlighted with stars of different colors, while those encoded by the human genome are indicated in green. (E) The co‐abundance network within Overweight group's CAG80. Edge width and color represent the strength and correlations between HQMAGs, and the positive correlations are highlighted in red. Stars depicted within the network denote enzymes annotated within the HQMAGs. (F) Repeated‐measures correlation between 4‐Pyridoxic acid and continuous glucose monitoring (CGM) metrics. Red indicates a positive correlation, and blue indicates a negative correlation. Asterisks indicate significance levels as follows: ^+^indicates *p* < 0.1, *indicates *p* < 0.05, **indicates *p* < 0.01. MBG: mean blood glucose; Estimated A1C: estimated glycated hemoglobin level; GMI: glucose management indicator; SD: standard deviation; Daytime: 6 am to 10 pm; Nighttime: 10 pm to 6 am; % Time < 180 mg/dL: the percentage of time spent with glucose levels < 180 mg/dL; TAR: time above range, the percentage of time spent with glucose levels > 180 mg/dL; TIR: time in range, the percentage of time spent with glucose levels between 70 and 180 mg/dL; TBR: time below range, the percentage of time spent with glucose levels < 69 mg/dL; MAGE: mean amplitude of glycemic excursion; CONGA 1 h: continuous overlapping net glycemic action for 1 h; LBGI: low blood glucose index; HBGI: high blood glucose index; LAGE: largest amplitude of glycemic excursions; CV: coefficient of variation.

Procrustes analysis confirmed alignment between fecal metabolites profiles and microbiota composition (Figure S9). In the Overweight group, 25 of 45 significant time‐delayed associations suggested potential effects of CAGs on metabolites (Figure 5A). Notably, CAG80 was positively correlated with 4‐Pyridoxic acid (4‐PA), a major vitamin B6 catabolite (local similarity score = 0.760; Delay = 3; false discovery rate = 0.044), with both exhibiting sustained increases from the intervention onset (Figure 5B,C), noted in most participants (Figure S10A,B). All seven HQMAGs in CAG80 possessed genes encoding enzymes essential for 4‐PA metabolism (Figure 5D,E). Additionally, 4‐PA was significantly negatively correlated with mean blood glucose (MBG), glucose management indicator, night SD, and J Index (Figure 5F).

**FIGURE 6:**
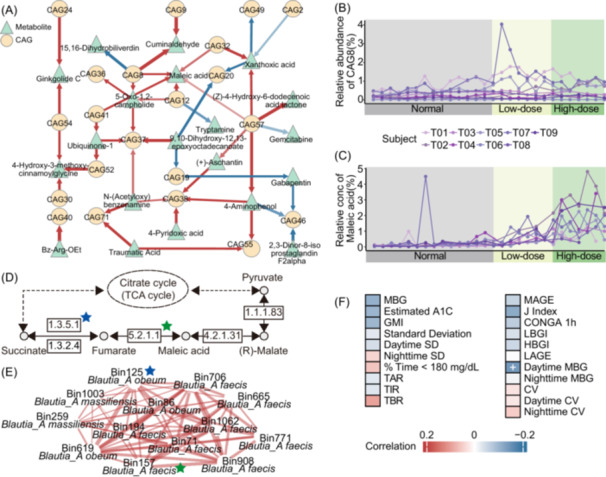
Role of maleic acid in the association between gut microbiota and host glycemic stability in the T2D group. (A) Significant time‐delayed correlations between CAGs and fecal metabolites. Changes in CAG8 (B) and maleic acid (C) in the T2D group during the trial. (D) The KOs in butanoate metabolism (KEGG pathway #00650) predicted in T2D group's CAG8 are marked with stars of different colors. (E) The co‐abundance network within T2D group's CAG8. Stars depicted within the network denote enzymes annotated within the HQMAGs. (F) Repeated‐measures correlation between maleic acid and CGM metrics.

Similarly, in the T2D group, 25 of 47 significant time‐delayed associations indicated the effects of CAGs on metabolites (Figure 6A). CAG8 was positively correlated with maleic acid (local similarity score = 0.729; Delay = 1; false discovery rate = 0.046), both showing rapid increases during intervention (Figures 6B,C, S10C,D). Two HQMAGs within CAG8, from *Blautia obeum* and *Blautia faecis*, harbored genes for key enzymes in maleic acid metabolism (Figure 6D,E), while maleic acid was negatively correlated with daytime MBG (Figure 6F). These findings suggested that dietary fiber‐responsive gut microbiota may improve glycemic control through specific metabolite modulation.

### Serum metabolite mediation effect in microbiota‐induced host metabolic improvement

**FIGURE 7:**
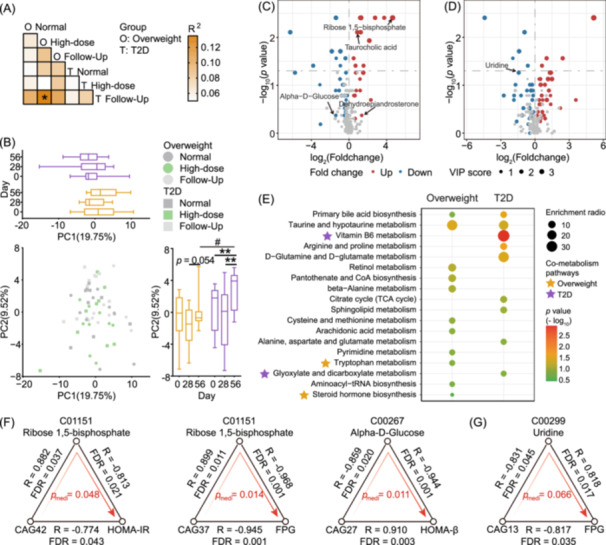
Significant changes in serum metabolome following dietary fiber intervention. (A) PERMANOVA test based on Euclidean dissimilarity with 999 permutations, BH‐adjusted *p* < 0.05. (B) PCA of serum metabolite levels (Euclidean dissimilarity), and the dots indicate samples. Across groups, Mann–Whitney *U* test (two‐tailed) was used for intergroup comparisons on Day 0, while one‐way ANCOVA was used on Day 28 and Day 56, adjusted by the Day 0 values (^#^indicates *p* < 0.05). Within each group, Wilcoxon matched‐pairs signed‐rank test (two‐tailed) was performed to evaluate the differences at various intervention time points compared to the values of Day 0 (*indicates *p* < 0.05 and **indicates *p* < 0.01). Differential metabolites in serum identified between pre‐ and post‐intervention in the Overweight group (C) and the T2D group (D) are represented by volcano plots, with those having a variable importance in the projection (VIP) ≥ 1 highlighted in red and blue. (E) The pathway enrichment analysis of serum differential metabolites by KEGG in the Overweight group and T2D group after dietary fiber intervention. Asterisks highlight metabolic pathways concurrently enriched in both fecal and serum metabolomes. The mediation effects of serum metabolites that were significant at *p* < 0.05 in the Overweight group (F) and *p* < 0.1 in the T2D group (G). Shown are microbiome (left), serum metabolites (middle), and glycometabolism parameters (right). The black lines indicate the associations between the two factors, with corresponding repeated‐measures correlation coefficients and BH‐adjusted *p* values. Direct mediation and corresponding *p* values are shown in red.

Serum metabolomic analysis revealed modest changes post‐intervention (Day 28) and at follow‐up (Day 56, Figure 7A,B), with 23 and 34 differential metabolites observed in the Overweight and T2D groups, respectively (Figure 7C,D). Taurine and hypotaurine metabolism, as well as primary bile acid biosynthesis, were enriched in both groups (Figure 7E). Several pathways were enriched in both fecal and serum profiles, with steroid hormone biosynthesis and tryptophan metabolism in the Overweight group, and vitamin B6, glyoxylate, and dicarboxylate metabolism in the T2D group.

Mediation analysis revealed six significant linkages in the Overweight group (Figure 7F and Table S16). Ribose 1,5‐bisphosphate (Rib‐1,5‐P2) was found to mediate the impact of CAG42 on HOMA‐IR and CAG37 on FPG. Functional gene analysis showed that both CAG42 (dominated by *Bifidobacterium pseudocatenulatum*) and CAG37 (dominated by *Fusicatenibacter saccharivorans*) could synthesize Rib‐1‐P, an upstream metabolite of Rib‐1,5‐P2, via the pentose phosphate pathway. Additionally, Alpha‐d‐Glucose mediated the impact of CAG27 on HOMA‐beta. CAG27, dominated by species from *Faecalibacterium*, possessed the genomic capacity to metabolize Alpha‐d‐Glucose through multiple glucose metabolism pathways. In the T2D group, uridine potentially mediated the impact of CAG13 on FPG (*p* < 0.1, Figure 7G and Table S17). CAG13, primarily consisting of *Blautia_A* species, showed genomic capacity for uridine metabolism via ABC transporters and pyrimidine metabolism pathways. In summary, these findings provided insights into the potential interactions among microbiota, serum metabolites, and host metabolism.

## DISCUSSION

Our study underscores the necessity of high‐frequency sampling in gut microbiota dynamics research, which is often overlooked by conventional sampling approaches. Using dietary fiber as an effective perturbation, we integrated daily sampling, guild‐level, and time‐series analyses to reveal distinct response patterns among various microbiota members linked to varied genetic capacities for carbohydrate metabolism. Time‐delayed analysis of longitudinal multi‐omics data revealed specific metabolites that potentially mediate interactions between fiber‐responsive gut microbiota and host metabolic improvement. Overall, our comprehensive framework provides reliable targets for mechanistic investigations and lays a foundation for precision therapeutic interventions.

High‐frequency sampling demonstrated significant advantages for accurately characterizing microbial dynamics in our study. Regarding the identification of microbial dynamics, our comparisons revealed that relying solely on pre‐ and post‐intervention snapshots might be misleading [3, 18]. For example, while pre‐to‐post comparison suggested decreases in CAG5 and CAG9 in the Overweight group, daily sampling data revealed significant early increases in these CAGs during the low‐dose phase. Such misidentification could overlook the potential biological significance of microbial members. Specifically, *Mediterraneibacter faecis* in CAG5 is known to play a critical role in polysaccharide degradation and SCFA synthesis [23, 41]. Similarly, the dominant species in CAG9, *Anaerobutyricum soehngenii*, contributes to the enhancement of insulin sensitivity in overweight and insulin‐resistant subjects [26]. These findings suggest that conventional sampling approaches may miss critical transient microbial dynamics with functional relevance.

High‐frequency sampling enables the identification of time‐delayed associations among multi‐omics data. Using local similarity analysis, we identified two novel potential interactions among gut microbiota, metabolites, and host metabolism. Regarding 4‐PA found in the Overweight group, a previous study in ob/ob mice demonstrated its ability to enhance glucose metabolism [42]. Similarly, the potential metabolic benefit of maleic acid found in the T2D group aligns with findings from Zucker diabetic fatty rats, where cecal concentrations of maleic acid were negatively correlated with plasma alanine transaminase (ALT) levels [43]. Collectively, these observations indicate that high‐frequency sampling can provide more accurate and reliable targets for mechanistic investigations and the development of novel therapeutic interventions.

Strain‐level analysis is essential for accurately characterizing microbial responses to dietary interventions. Comparisons with similar studies revealed distinct temporal dynamics of *Bacteroides* between human and animal models, despite their shared characteristic of rapid response to dietary interventions. In our human study, *Bacteroides uniformis* was the predominant species in CAG25 in the Overweight group, and in CAG6 and CAG7 in the T2D group. The abundance of these CAGs decreased during dietary fiber intervention. In contrast, animal study reported a significant increase in *B. uniformis* in response to inulin [3]. These discrepancies likely reflected genomic variations within the same species across different hosts, implying the strain‐specific nature of microbial responses. Therefore, future research should focus on detailed strain‐level investigations rather than solely on the genus or species levels to capture adequate microbial dynamics.

Guild‐based clustering is a robust approach for understanding interactions among gut microbiota members. By focusing on stable inter‐member interactions, this approach partially addresses the limitations of conventional taxon‐based and gene‐centric methods. The effectiveness and broad applicability of this approach have been demonstrated across multiple studies, where functionally coherent microbial groups identified showed significant associations with multiple diseases, including obesity [44], type 2 diabetes [45], and COVID‐19 [46]. The consistent observation of “seesaw” structures between competing guilds across diseases further highlights its utility in disease classification and intervention assessment [15]. Notably, our study enhances the reliability of CAG clustering by capturing true physical coexistence among members. We effectively reduced spurious links caused by interindividual variation by constructing individual microbial networks and retaining correlations consistently identified in at least six subjects per group. The biological relevance of our clustering was further supported by functional analysis, which revealed distinct CAZyme profiles and phosphotransferase system‐related KO signatures among CAGs with different temporal dynamics. Additionally, the significant associations between CAG dynamics, metabolite changes, and host glycemic variability provide independent biological validation for our clustering method. Collectively, these findings demonstrate that incorporating physical coexistence improves the reliability of CAG‐based analysis.

Building on the methods discussed above, we revealed that similar temporal response patterns among microbial members were genetically determined. Similarities in functional genes indicate comparable metabolic functions. With respect to carbohydrate metabolism, CAGs with increased abundance were enriched in CAZy genes related to the utilization of inulin and sucrose/fructans, which were contributed by enzymes from four glycoside hydrolase (GH) families [47]. Specifically, the GH32 family includes glycoside hydrolases that hydrolyze the glycosidic bonds of fructans [48]. Inulin fructotransferase from the GH91 family is able to convert inulin to difructose anhydride III [49]. Meanwhile, the GH70 family can synthesize α‐glucans from sucrose and starch [50], and the GH68 family preferentially uses sucrose as the substrate to synthesize fructans and fructooligosaccharides [48]. In terms of transport, increased CAGs demonstrated enhanced β‐glucoside transport capabilities. The EIIBCA component of the PTS, encoded by *bglF* and *bglP*, is responsible for the transport and phosphorylation of extracellular β‐glucoside. A previous study demonstrated that deficiency in these transport components significantly impaired the utilization of various sugars, including β‐glucoside, sucrose, and fructose, highlighting their crucial role in transport [51]. However, it is important to note that the proportion of genes does not necessarily reflect actual enzyme activity within bacteria. Thus, comprehensive multi‐omics analysis and experimental validation are essential to determine the metabolic characteristics of microbiota members.

### Limitations and future directions

Our study has several limitations. While our cohort size is relatively small compared to many clinical trials, our within‐subject design with daily sampling has revealed distinct microbiota dynamics that were overlooked by conventional approaches. External validation for our findings is challenging due to limited comparable datasets. Moreover, temporal associations alone cannot establish causality without further experimental studies. As part of our future prospects, we plan to conduct large‐scale population studies across multiple centers with high‐frequency sampling to enhance reproducibility and the generalizability of our findings.

## CONCLUSION

In conclusion, our study demonstrates the substantial value of high‐frequency sampling in capturing the dynamic responses of gut microbiota, which may be missed by pre‐to‐post comparisons. By identifying associations between microbiota and metabolites that are beneficial to host health, this approach further offers targets for causal relationship investigations and enhances the clinical application of microbiota and metabolites. Future research could explore its application across various diseases, thereby enhancing our understanding of microbiota dynamics.

## METHODS

### Study registration

The clinical trial, conducted at the Shanghai Pudong Hospital (Shanghai, China) and Shanghai General Hospital (Shanghai, China) between August and October 2021, aimed to identify the characteristics of gut microbiome changes in overweight participants with and without T2D during dietary fiber intervention. The study protocol was approved by the ethics committee of the Chinese Ethics Committee of Registering Clinical Trials (ChiECRCT20210194, June 20, 2021). The study was conducted according to the principles of the Declaration of Helsinki and registered in the Chinese Clinical Trial Registry (ChiCTR2100046478). All participants provided written informed consent.

Inclusion and exclusion criteria. A total of 23 participants with obesity or overweight (BMI 24.0–35.0 kg/m^2^, body weight changes < 15% over past 3 months), aged 25–60 years, blood pressure < 180/110 mmHg, and triglyceride < 8 mmol/L were screened. Participants who met the criteria for determining T2D based on the physical examination report or medical records of the past 6 months were assigned to the T2D group. The criteria for determining diabetes mellitus include either HbA1c ≥ 6.5%, with the testing method certified by the United States National Glycohemoglobin Standardization Program and strictly following the procedures established by the Diabetes Control and Complication Trial conducted in the United States, or a random blood glucose at any point ≥ 11.0 mmol/L.

Comprehensive medical evaluation included medical history, food allergies assessment, physical examination, OGTT, and various blood tests. Exclusion criteria included: (1) Pregnancy, lactation, or plans to become pregnant; (2) Diagnosed diabetes for more than 2 years; or less than 2 years with complications, or with hypoglycemia; (3) Severe infections, major trauma, surgery, or other acute stress conditions; chronic gastrointestinal issues in the past month; (4) Use of thiazolidinediones, glucosidase inhibitors, weight loss drugs, corticosteroids, drugs affecting gastrointestinal motility, or post‐transplant drugs in the last 3 months; (5) Chronic kidney disease or abnormal levels of blood urea nitrogen or creatinine; chronic liver disease or high ALT or aspartate aminotransferase (AST) levels; high blood or urine amylase levels; (6) Severe or unstable angina, coronary insufficiency, or heart failure classified as NYHA Class III or IV; diabetic peripheral neuropathy with pain, urinary or gastric retention, foot ulcer, or urgent retinopathy requiring immediate care; (7) Drug or alcohol addiction in the past year; (8) Any condition affecting study participation or evaluation as determined by researchers.

Our study was not a randomized controlled trial, but rather employed a within‐subject study design. The primary aim of this study was not to assess significant changes in primary and secondary outcomes before and after dietary fiber intervention. Instead, it aimed to investigate the dynamic changes in participants' gut microbiota composition during the trial. Therefore, specific sample size determination was not applicable to this study. We initially planned to recruit 8 to 20 overweight participants per group, evenly split between those with and without T2D. Out of 23 screened participants, 20 were enrolled (*n* = 10 in the Overweight group, *n* = 10 in the T2D group). Among them, one T2D group participant withdrew early, and one Overweight group participant had once reported mild diarrhea and continued consuming dietary fiber supplements.

### Study design

Study design and flow are detailed in Figures 1, S1A, respectively. Participants underwent a 2‐week dietary fiber intervention, consuming 18 g/day in the low‐dose phase (Day 14–Day 20) and 36 g/day in high‐dose phase (Day 21–Day 27) before meals, while maintaining their usual diet. During the low‐dose phase, participants took 6 g of insoluble fiber before breakfast and 6 g of mixed fibers (3 g insoluble, 3 g soluble) before both lunch and dinner. Fiber dose doubled in the high‐dose phase. This was followed by a 4‐week follow‐up. T2D group participants also maintained physician‐prescribed antidiabetic therapies throughout the study (Table S3). Primary outcome was the percentage of time per day within target glucose range (TIR, 70–180 mg/dL); Secondary outcomes included dynamic glucose excursion, blood lipid profiles (cholesterol, triglycerides, LDL, and high‐density lipoprotein), blood pressure, and BMI.

### Dietary fiber supplement

Participants received dietary fiber supplements on Day 14, prepared by Shanghai Adfontes Technology Co., Ltd., and tested by SGS‐CSTC Standards Technical Services Co. Ltd. to confirm nutritional content and safety against microbial and pathogenic contamination. Insoluble dietary fiber supplements included wheat bran, oat bran, coix seed, buckwheat, highland barley, chia seeds, quinoa, rye, foxtail millet, and monk fruit extract. Soluble dietary fiber supplements contained resistant dextrin, inulin, fructooligosaccharides, and xylooligosaccharides. Participants were advised to take these supplements with water. Nutritional details are in Supplementary Table S2.

### Blood sample collection

Blood sample collections were conducted on Day 0, Day 28, and Day 56 in Shanghai Pudong Hospital; samples were left at room temperature for 30 min, then centrifuged at 2500 g for 15 min at 4 ± 2°C to separate serum, which was used for clinical measurements and lab storage. Venous blood was analyzed for glycemic traits, blood routine examination, and blood biochemical examination in Shanghai General Hospital.

### Fecal sample collection

Fecal samples were self‐collected daily during the normal diet and dietary fiber intervention phases using provided containers. Samples were immediately transported on ice to the laboratory for quality assessment and storage at −80°C. On Day 56, samples collected post‐waking were immediately placed on dry ice, brought to the hospital, and then transported to the lab for freezing at −80°C.

### Anthropometry data collection

Physical examinations were conducted on Day 0, Day 14, Day 28, and Day 56 in Shanghai Pudong Hospital. Heights and body weights were measured using a digital scale with a stadiometer to an accuracy of 0.1 cm and 0.1 kg, respectively. Waist circumference was measured between the lower rib and the iliac crest, and hip circumference was measured at the widest part of the buttocks using a cloth tape to the nearest 0.1 cm. Blood pressure (systolic blood pressure, SBP; diastolic blood pressure, DBP) was taken using an OMRON HEM‐7200 digital electronic sphygmomanometer after participants had rested quietly for 15 min. BMI was calculated as kg/m^2^.

### Continuous glucose monitoring

A CGM sensor was placed on participants' upper left arm on Day 0 (Abbott FreeStyle Libre), recording glucose data every 15 min for 14 days. Sensors were replaced on Day 14 and removed on Day 28. Variables, like TIR, MAGE, and CONGA were calculated using R package cgmanalysis [52]. Due to the potential impacts of placing, replacing, and removing sensors on data accuracy, and the possibility that fasting on Day 28 might also affect CGM measurements, data from the days of sensor manipulation were excluded from analysis.

### Laboratory measurement

HbA1c levels were measured by a G8 automated glycohemoglobin analyzer (Tosoh Bioscience). Plasma glucose, total cholesterol, total bilirubin, high‐density lipoprotein, LDL, AST, ALT, microalbumin, and urine creatinine were measured by ADVIA Chemistry XPT System (Siemens Healthineers). Serum insulin was determined using the ADVIA Centaur XP system (Siemens Healthineers).

### Statistical analysis of clinical data

Statistical analysis was performed in the R environment (R version 4.1.2). For the change of standardized CGM metrics, the Mann–Whitney test (two‐tailed) was used for intergroup comparisons during the normal diet phase, while the one‐way ANCOVA was used for intergroup comparisons throughout the dietary fiber intervention phase, adjusted by the normal diet phase mean value. In the Overweight group, *n* = 9 for most analyses and *n* = 8 for high blood glucose index. In the T2D group, *n* = 9 (Normal), *n* = 8 (low‐dose and high‐dose). For the percent changes in physical examination measurements and test results, the Mann‐Whitney test (two‐tailed) was used for intergroup comparisons at the same time point. Moreover, the Wilcoxon matched‐pairs signed‐rank test was utilized for intra‐group comparisons over different time points for two types of measurements (one‐tailed for CGM metrics, two‐tailed for physical examination metrics). Significance was determined as *p* < 0.05. In the Overweight group, *n* = 10 for most comparisons, and for SBP and DBP, *n* = 9 at Day 14, for SBP and DBP, *n* = 8 on Day 28; for waist circumference (WC), hip circumference (HC), and waist‐hip ratio (WHR), *n* = 9 on Day 28. In the T2D group, *n* = 9 for all analyses.

### Metagenomic sequencing

A total of 443 fecal samples were collected. Genomic DNA was extracted using the QIAamp PowerFecal Pro DNA Kit (QIAGEN, 51804) and quantified with a Qubit 3.0 fluorometer (Thermo Fisher, Q33217) using the Qubit dsDNA HS Assay Kit (Thermo Fisher, Q32854). DNA integrity was assessed on 1% agarose gels. For library preparation, 300 ng of DNA was enzymatically fragmented with KAPA Frag Enzyme and KAPA Frag buffer (KAPA Biosystems, KK8514), followed by end repair and A‐tailing at 65°C for 30 min. Adapters were ligated after adding adapter stock, ligation buffer, and DNA ligase, and incubated for 15 min at 20°C. Libraries were cleaned and size selected with VAHTS DNA Clean Beads (Vazyme, N411‐03). Post‐amplification, libraries underwent QC, including size analysis by Agilent 2100 High Sensitivity DNA kit (Agilent, 43513) on an Agilent 2100 bioanalyzer (Agilent, G2939BA), and quantification using the StepOnePlus RT PCR System (Thermo Fisher, 4376600).

Shotgun sequencing was conducted using the Illumina Novaseq 6000 platform (Illumina), producing paired‐end 150 bp reads. Sequencing for 433 samples generated a total of 26.84 billion raw reads, averaging 62.00 million paired‐end raw reads per sample. Sequencing of Overweight group samples generated 13.81 billion raw reads, averaging 62.21 million reads per sample. For the T2D group, 13.03 billion raw reads were generated, averaging 61.76 million reads per sample.

### Data quality control

Trimmomatic v0.39 [53] was used to preprocess the raw reads into clean reads by removing low‐quality reads containing over 35 bp of “N” bases (quality ≤ 15) and reads with adapter sequences exceeding 10 bp. On average, 1.91% of reads were filtered out in the Overweight group, and 1.72% of reads were filtered out in the T2D group. Detailed counts of raw and clean reads per sample are listed in Table S5. Clean reads were aligned to the human genome reference database GRCh38 using Bowtie2 v2.4.1 software [54] with parameters: ‐‐end‐to‐end, ‐‐more‐sensitive, ‐I 200, ‐X 500. The average host contamination rate was 0.10% in the Overweight group and 0.08% in the T2D group.

### De novo assembly and binning

High‐quality paired‐end reads were de novo assembled into ≥ 500 bp contigs using MEGAHIT v1.2.9 individually for each sample [55]. In the Overweight group, 21,401,340 contigs (average N50 = 10,564, max length = 844,713 bp) were obtained. In the T2D group, 16,567,176 contigs (average N50 = 12,548, max length = 1,329,489 bp) were obtained. For binning, high‐quality reads were mapped to the contigs using Bowtie2 v2.4.1 (‐‐very‐sensitive‐local), and the output SAM file was indexed using Samtools v0.1.19 [56], then ranked, and converted into a BAM file. Depth values were computed from the BAM files using the jgi_summarize_bam_contig_depths tool of MetaBAT2 v2.15.2 [57], applying parameters of‐‐percentIdentity 0.97, ‐‐minContigLength 1000, and ‐‐minContigDepth 1.0. The assembled contigs were binned with MetaBAT2 v2.15.2 (‐‐minContig 1500) for each sample. To increase bin completeness, contigs from all the samples were combined, and MetaBAT2 was used to reassemble them into bins according to the tetranucleotide frequency and abundance distance probability (ADP). MAGs were quality‐controlled using CheckM v1.1.2 [58]. Mash v2.2.2 [59] was used to build the hierarchical clustering tree of MAGs with a threshold of 0.25 for distance relationship and branching cluster. MAGs were further dereplicated using dRep v3.2.0 [60], with parameters: ‐pa 0.9 (primary cluster at 90%), ‐sa 0.99 (secondary cluster at 99%), and ‐con 5 (contamination threshold of 5%). A total of 1100 high‐quality metagenome‐assembled genomes (HQMAGs, integrity > 75%, contamination < 10%) were selected for strain‐level analysis.

### Abundance calculation and taxonomic assignment

Taxonomic assignment of HQMAGs was performed by using GTDB‐Tk v1.3 [61] (Genome Taxonomy Database Toolkit) and GTDB R95 reference database (released in July 2020). Bowtie2 v2.4.1 was used to align clean reads to HQMAGs to obtain read abundance. The relative abundance of each genome was defined as the number of reads aligning to each HQMAG, normalized by the genome size.

### Gut microbiome functional analysis

The PRODIGAL v2.6.3 [62] software was used to predict genes for HQMAGs separately. The predicted coding genes were annotated and compared with KEGG (2019.10) and CAZy (2020) to obtain the functional information of the genome. Detailed steps are as follows: (1) DIAMOND v0.9.9.110 software was used to compare the gene ORF protein sequences predicted by each HQMAG with various functional databases (blastp, e‐value ≤ 1e‐5); (2) Filtering of comparison results: For the comparison results of each sequence, the comparison result with the highest score (one HSP > 60 bits) was selected for subsequent analysis.

### Gut microbiome network construction and analysis

Prevalent HQMAGs, present in more than 45% of each participant's samples, were utilized to build co‐abundance networks for each one. FastSpar was employed to calculate genomic correlations using 1000 permutations based on HQMAG abundances in each participant [63]. Only correlations with *p* ≤ 0.05 were considered for subsequent analysis. Significant correlations (positive/negative) across ≥ 6 participants within the group were included in the group's co‐abundance network. Group co‐abundance networks were visualized using Cytoscape v3.10.1, while individual participant networks were displayed using Gephi v0.10.1. The correlation coefficient of each stable HQMAG pair was calculated as the average significant results within selected subjects in each group. The HQMAG correlation coefficient matrix was transformed into a distance matrix (1 ‐ correlation coefficient) and clustered using Ward.D2 hierarchical clustering method. PERMANOVA (9,999 permutations) was applied to determine distinct CAGs, with *p* < 0.001 and a minimum size threshold = 5 [13].

### Statistical analysis of microbiome data

For alpha and beta diversity analysis, statistical methods were consistent with CGM metrics. To compare the carbohydrate utilization and transport capacity across clusters exhibiting increasing, decreasing, or unchanged temporal patterns, we calculated the proportion of related encoding genes within each CAG by summing the numbers of these genes across HQMAGs constituting that CAG. The Mann‐Whitney test (two‐sided) was used for pairwise comparisons of CAZy gene and KOs proportion between the three cluster categories (increased, decreased, and unchanged clusters). The Kruskal–Wallis test with Dunn's post hoc was used to evaluate the difference among clusters. PERMANOVA was performed with Bray–Curtis distance on the microbiome at HQMAG level using the adonis2 function from the vegan v2.6‐4 (strata in subject, 999 permutations, BH adjust). Participant‐adjusted PCoA plots of the genomes based on Bray–Curtis distance were performed with aPCoA package [64]. LinDA v0.2.0 [65] was used to identify key CAGs with significant abundance shifts using linear mixed model (LMM). To assess changes over the entire intervention period compared to the normal diet phase, both participant and day were considered as random effects. To directly compare changes between the end of the intervention (Day 27) and the end of the normal diet (Day 13), only the participant was regarded as a random effect. To assess temporal changes in CAG abundance among participants within each group during the dietary fiber intervention phases, LMM was performed using the lmer function from the R package lme4 v1.1‐35.1: lmer(log(CAG)~ day + (1 | participant)). This model analyzed both the daily data from the dietary fiber intervention phase and the average abundance observed during the normal diet phase. Changes in daily CAG abundance were calculated by summing each day's fixed effect coefficient with the intercept, which represents the average CAG abundance during the normal diet phase. Dynamic time warping (DTW) distance between CAGs temporal dynamics was calculated using the dtwdist function from the R package dtw v1.23‐1 [66]. The optimal number of clusters was determined based on the similarity of temporal abundance changes and the silhouette widths. *n* = 10 in Overweight group and *n* = 9 in T2D group for analyses.

### Fecal and serum sample preparation for metabolome analysis

Fecal sample (100 mg) was combined with 100 mg glass bead and 600 µL of methanol (−20°C, containing isotope‐labeled standards), followed by grinding and ultrasonication. The mixture was centrifuged (12,000 rpm, 4°C, 10 min) and filtered through a 0.22 μm membrane. Equal amounts of supernatant was pooled for quality control. Serum samples (100 µL) were thawed at 4°C, mixed with 400 µL methanol (−20°C) and 100 µL of isotope‐labeled standards, and then centrifuged under the same conditions as fecal samples. The supernatants were concentrated, dried, and then reconstituted in 150 µL of 80% methanol (−20°C), followed by centrifugation and filtration. 10–25 µL of each serum sample was pooled for quality control.

### Metabolite detection

LC analysis was performed on a Vanquish UHPLC System (Thermo Fisher Scientific). Chromatographic separation was performed by Acquity UPLC HSS T3 columns (2.1 × 150 mm, 1.8 μm; Waters) at 40°C and 0.25 mL/min flow rate. Metabolites were detected on Orbitrap Exploris 120 (Thermo Fisher Scientific) with an ESI. Simultaneous MS1 and MS/MS (Full MS‐ddMS2 mode, data‐dependent MS/MS) acquisition was applied with dynamic exclusion. Data were processed using Proteowizard and XCMS. Compound information (m/z, retention time, peak area) was exported. Chemicals were annotated by searching against mzCloud, LipidMaps, Human Metabolome Database, and a self‐developed PANOMIX Biomedical Tech database (Suzhou, China).

### Statistical analysis of metabolite

A total of 1194 fecal metabolites were identified and filtered using interquartile range (IQR) by MetaboAnalyst 6.0. Data normalization involved sum normalization, log‐transformed (base 10), and Pareto‐scaled. Differences in metabolomic profiles were assessed via PERMANOVA (Euclidean distance, 999 permutations). PCA was performed using the prcomp function from stats v4.1.2. Statistical and pathway enrichment analysis for differential metabolites was conducted using MetaboAnalyst [67] (v4.0, www.metaboanalyst.ca). Differences in fecal metabolites were identified within each subject. OPLS‐DA distinguished differential metabolites between dietary phases, and Mann–Whitney *U* test (two‐sided) assessed differences at the univariate level, defining significant metabolites as those with *p* < 0.05 and variable importance in the projection (VIP) ≥ 1. Due to insufficient sample sizes for O04 and T02, constructing the OPLS‐DA model for them was not feasible. For the remaining analyses, *n* = 10 in Overweight group and *n* = 9 in T2D group.

Serum metabolites were similarly analyzed, with VIP ≥ 1 indicating differential metabolites. In the Overweight group, *n* = 10 on Day 0 and Day 14, and *n* = 9 on Day 56. In the T2D group, *n* = 9 across all time points.

### Procrustes analysis

Procrustes analysis was then performed between the aPCoA of HQMAGs and the PCA of metabolomics data to identify concordance between the two datasets using the procrustes function from the R package vegan v2.6‐4.

### Extended local similarity analysis

For assessing the dynamic trends of CAG abundance or fecal metabolite concentration over the 28‐day study period, previously described LMM was applied again. This analysis incorporated the entire 28‐day data, and the intercept was set to represent the average of CAG abundance or fecal metabolite concentration on Day 0. The time‐delayed correlations between CAG abundance and fecal metabolite concentrations over the 28‐day study period were calculated using the lsa_compute function (‐n percentileZ) of the Extended Local Similarity Analysis (ELSA v1.0.2) [68]. *p‐*value estimation was calculated using theoretical approximation (‐p theo), and only correlations with statistically significant BH‐adjusted *p* < 0.05 were retained for further analysis. Time‐delayed correlations between CAGs and fecal metabolites were visualized with Gephi v0.10.1. Repeated measure correlation was used to calculate the correlations between continuous glucose monitoring parameters and fecal metabolites using R package rmcorr v0.6.0 [69].

### Mediation analysis

Correlations among CAGs, serum metabolites, and glycemic traits were checked by repeated measure correlation analysis. Only pairs demonstrating significant pairwise correlations (BH‐adjusted *p* < 0.05) were included in the mediation analysis. Due to the correlation of samples within participants, LMM with participants as random intercepts was employed instead of the linear regression model. Causal mediation analysis was performed to infer the mediation effect of serum metabolites in the association between CAGs and glycemic traits, using ‐a mediate function from the R package mediation v 4.5.0.

## AUTHOR CONTRIBUTIONS

**Xiaotong Lin**: Formal analysis; data curation; writing—original draft; writing—review and editing; visualization. **Chaoxun Wang**: Resources; supervision; project administration; funding acquisition. **Biao Liu**: Resources; investigation. **Yin Zhu**: Resources; investigation. **Rui Zhai**: Investigation; resources; formal analysis. **Chenhong Zhang**: Conceptualization; writing—review and editing; supervision; project administration; methodology; funding acquisition.

## CONFLICT OF INTEREST STATEMENT

Chenhong Zhang is the cofounder of Adfontes (Shanghai) Co., Ltd. The remaining authors declared no conflict of interest.

## ETHICS STATEMENT

The ethics application (No. ChiECRCT20210194) was approved by the ethics committee of the Chinese Ethics Committee of Registering Clinical Trials.

## Supporting information


**Figure S1.** Dietary fiber intervention rapidly enhances glucose stability in overweight subjects with and without T2DM.
**Figure S2.** Participant factor significantly influenced gut microbiome diversity.
**Figure S3.** Rapid and significant changes in gut microbiota composition following dietary fiber intervention.
**Figure S4.** Co‐occurrence network construction method.
**Figure S5.** Co‐abundance network in overweight participants.
**Figure S6.** Co‐abundance network in overweight participants with T2DM.
**Figure S7.** Influence of participant factor on the fecal metabolite profile.
**Figure S8.** Volcano plots of fecal metabolite changes and enriched pathways.
**Figure S9.** Similarity in the changes in gut microbiota structure and fecal metabolome characteristics during the trial.
**Figure S10.** Temporal changes in individual CAG and fecal metabolite levels during the trial.

**Table S1.** Clinical parameters during the intervention.
**Table S2.** Nutritional composition of dietary fiber supplement.
**Table S3.** Antidiabetic medication use information for T2D subjects.
**Table S4.** Continuous glucose monitoring parameters during the intervention.
**Table S5.** Raw and high‐quality reads of each sample.
**Table S6.** Genome quality.
**Table S7.** Topological parameters of networks.
**Table S8.** Genomes taxonomic assignment by GTDB‐TK and clustering results in the Overweight group.
**Table S9.** Genomes taxonomic assignment by GTDB‐TK and clustering results in the T2D group.
**Table S10.** Dominant species within each CAG in the Overweight group.
**Table S11.** Dominant species within each CAG in the T2D group.
**Table S12.** Differences in the proportion of CAZyme genes among increased clusters in the Overweight group.
**Table S13.** Differences in the proportion of KOs related to carbohydrate transportation among increased clusters in the Overweight group.
**Table S14.** Differences in the proportion of KOs related to carbohydrate transportation among increased clusters in the T2D group.
**Table S15.** Differences in the proportion of KOs related to carbohydrate transportation among decreased clusters in the Overweight group.
**Table S16.** Co‐correlation relationships among gut microbiota, serum metabolites, and host metabolism in the Overweight group.
**Table S17.** Co‐correlation relationships among gut microbiota, serum metabolites, and host metabolism in the T2D group.

## Data Availability

The metagenomic sequencing data have been deposited under accession number PRJCA026382 and are publicly available (https://ngdc.cncb.ac.cn/bioproject/browse/PRJCA026382). The metabolomics data have been deposited in the OMIX database under accession number OMIX009789 (https://ngdc.cncb.ac.cn/omix/release/OMIX009789). The Methods section shows the parameters of the bioinformatic tools applied in the study. The data and scripts used are available at GitHub (https://github.com/linlin0026/imeta_1208/). Supplementary materials (figures, tables, graphical abstract, slides, videos, Chinese translated version, and update materials) may be found in the online DOI or iMeta Science http://www.imeta.science/.
