# Supplementary material for: Temporal response patterns of human gut microbiota to dietary fiber
Source: Imeta. 2025 May 10;4(4):e70046. doi: 10.1002/imt2.70046 (PMC12371261; doi:10.1002/imt2.70046)
Supplement: Supplementary file 1 — Figure S1. Dietary fiber intervention rapidly enhances glucose stability in overweight subjects with and without T2DM. Figure S2. Participant factor significantly influenced gut microbiome diversity. Figure S3. Rapid and significant changes in gut microbiota composition following dietary fiber intervention. Figure S4. Co‐occurrence network construction method. Figure S5. Co‐abundance network in overweight participants. Figure S6. Co‐abundance network in overweight participants with T2DM. Figure S7. Influence of participant factor on the fecal metabolite profile. Figure S8. Volcano plots of fecal metabolite changes and enriched pathways. Figure S9. Similarity in the changes in gut microbiota structure and fecal metabolome characteristics during the trial. Figure S10. Temporal changes in individual CAG and fecal metabolite levels during the trial. [file IMT2-4-e70046-s001.docx]

# Supporting information to

# Temporal response patterns of human gut microbiota to dietary fiber

Running title: Microbiota dynamics during dietary fiber intervention

Xiaotong Lin^1#^, Chaoxun Wang^2#^, Biao Liu^3^, Yin Zhu^3^, Rui Zhai^3^, Chenhong Zhang^1,4^ *

^1^State Key Laboratory of Microbial Metabolism, School of Life Sciences and Biotechnology, Shanghai Jiao Tong University, Shanghai 200240, China

^2^Department of Endocrinology, Shanghai Pudong Hospital, Fudan University Pudong Medical Center, Shanghai 201399, China

^3^Adfontes (Shanghai) Co., Ltd, Shanghai 200030, China

^4^Shanghai Jiao Tong University Sichuan Research Institute, Chengdu 610213, China

^#^These authors contributed equally: Xiaotong Lin, Chaoxun Wang
*Correspondence: [zhangchenhong@sjtu.edu.cn](mailto:zhangchenhong@sjtu.edu.cn) (Chenhong Zhang)


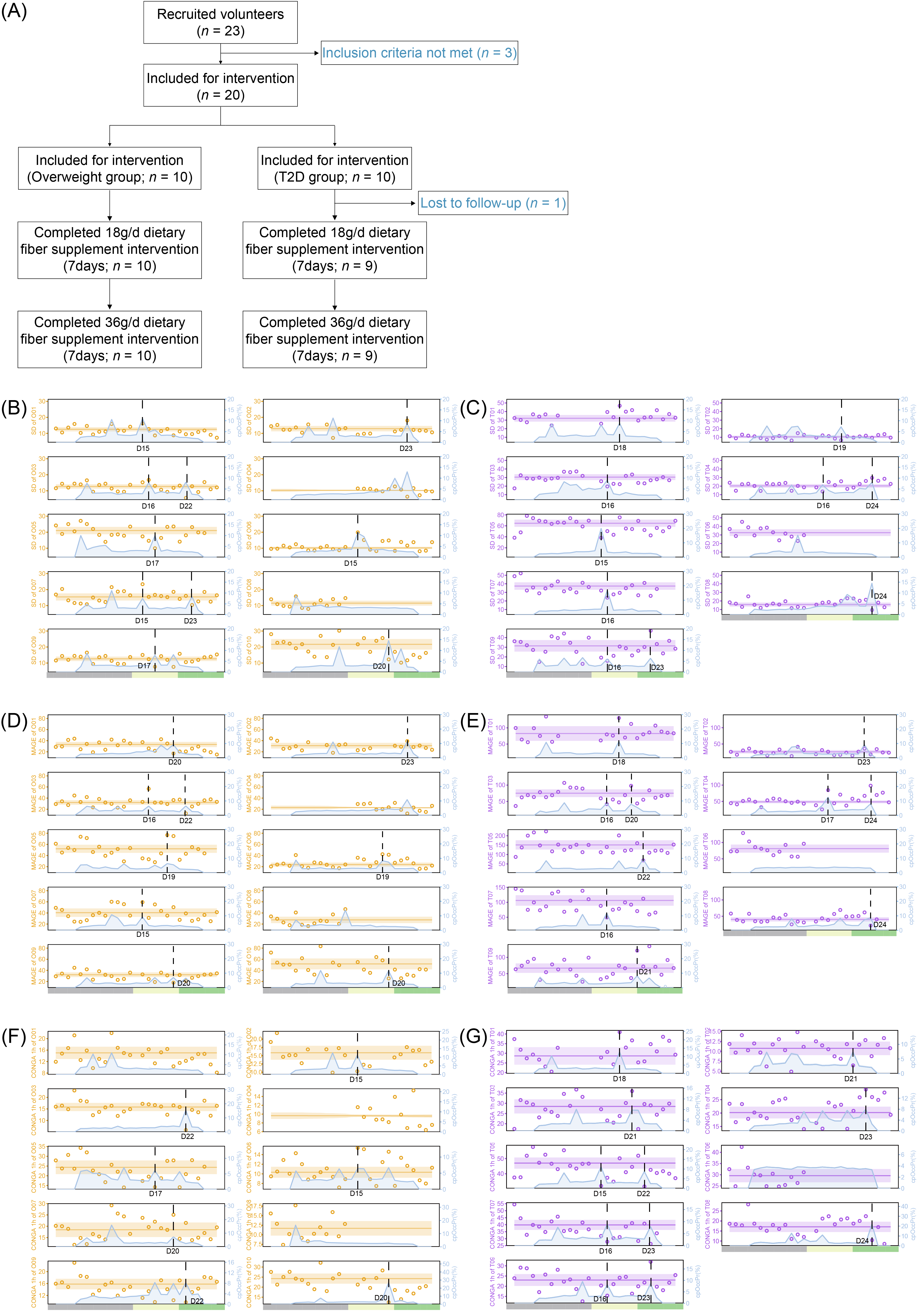
**Figure S1 Dietary fiber intervention rapidly enhances glucose stability in overweight subjects with and without T2DM.** (A) The CONSORT flow diagram. (B-G) Changepoints in Standard deviation (B and C), mean amplitude of glycemic excursion (D and E) and continuous overlapping net glycemic action (F and G) in each subject during the trial. Orange (Overweight) and purple (T2D) shaded regions illustrate the estimated interval ranges for CGM metrics, with solid lines indicating the average values during the normal diet phase. The blue curve indicates the probability of a changepoint (cpPr) occurrence throughout the trial. The normal diet phase (normal) is represented in grey, with low-dose dietary fiber intervention (low-dose) in light green, and high-dose dietary fiber intervention (high-dose) in green.


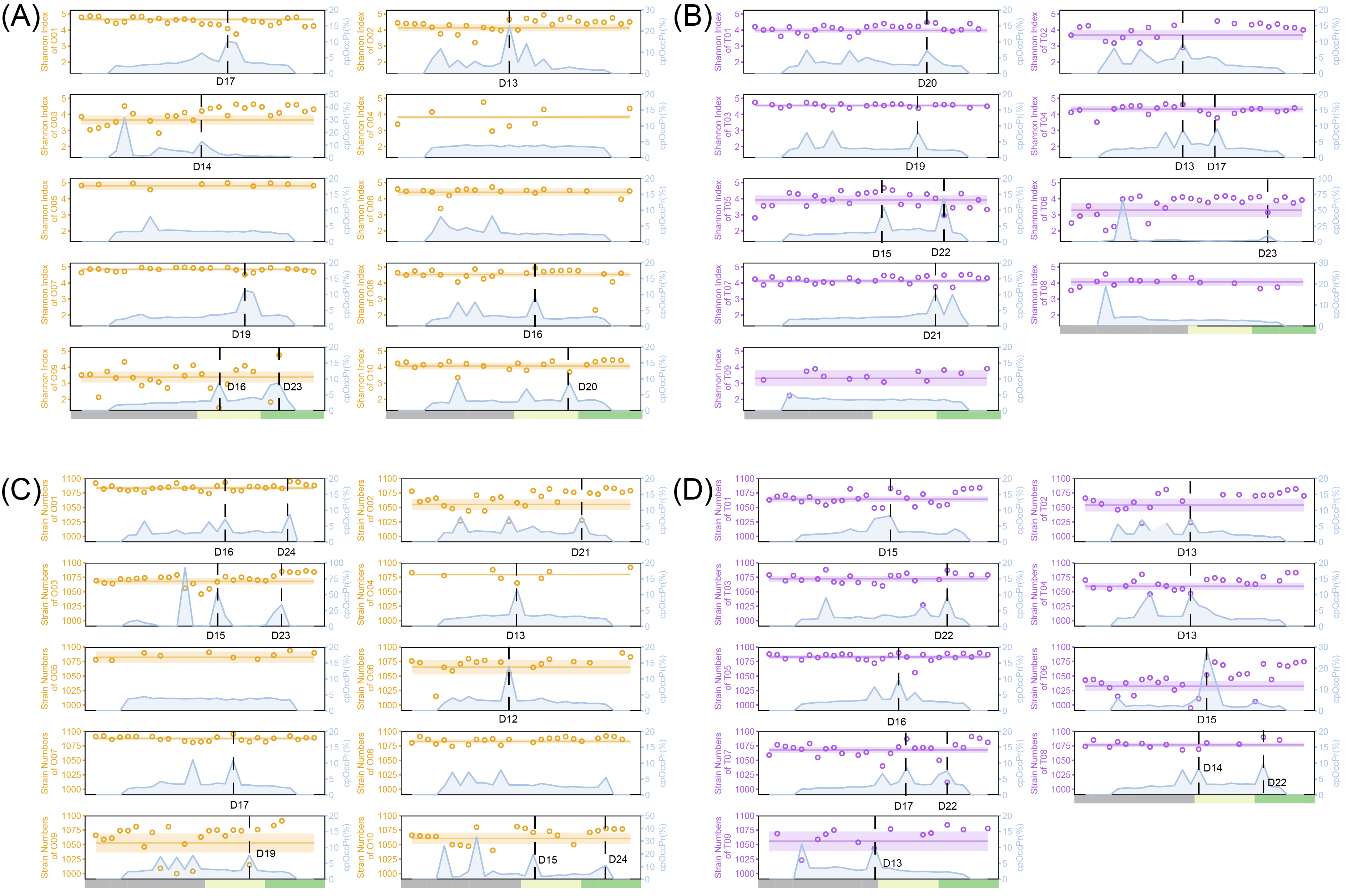
**Figure S2 Participant factor significantly influenced gut microbiome diversity.** (A-D) Changepoints of gut microbiota Shannon index (A and B) and strain numbers (C and D) for each subject throughout the trial.


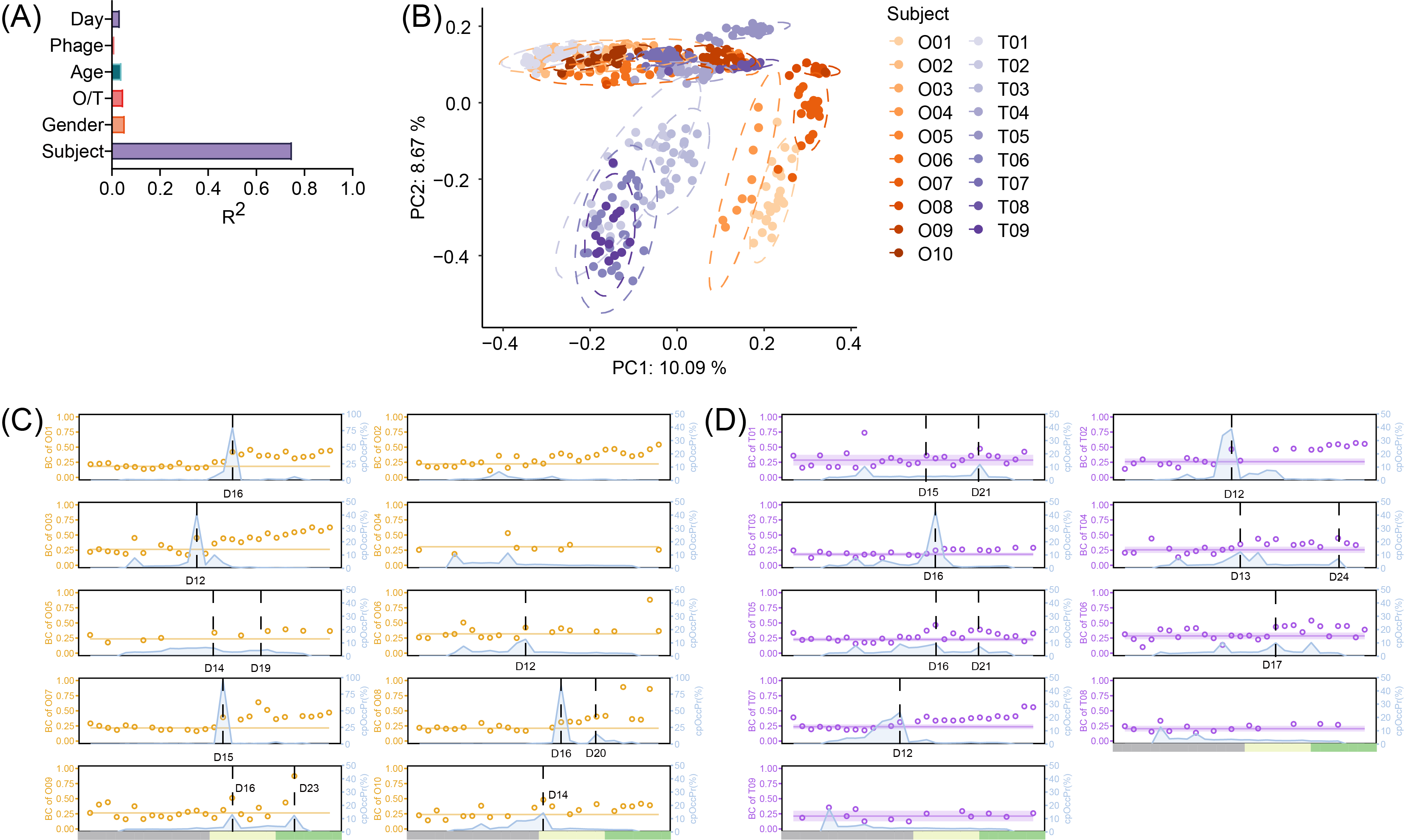
**Figure S3 Rapid and significant changes in gut microbiota composition following dietary fiber intervention.** Participant factor significantly influenced the gut microbiome structure. (A) PERMANOVA test based on Bray-Curtis dissimilarity with 999 permutations, BH-adjusted *p* < 0.05. (B) PCoA of Bray-Curtis dissimilarity at the Bins level, where individual dots represent samples. The color of each dot differentiates participants, highlighting individual microbiome diversity. (C and D) Changepoints of gut microbiota structure were identified for each subject throughout the trial. For comparison, the average microbiota abundance of each participant during the normal dietary phase was used as a baseline. The daily variation in microbiota structure from this baseline was quantified using Bray-Curtis distance. Orange (Overweight) and purple (T2D) shaded regions illustrate the estimated interval ranges for distance, with solid lines representing the average values during the normal diet phase.


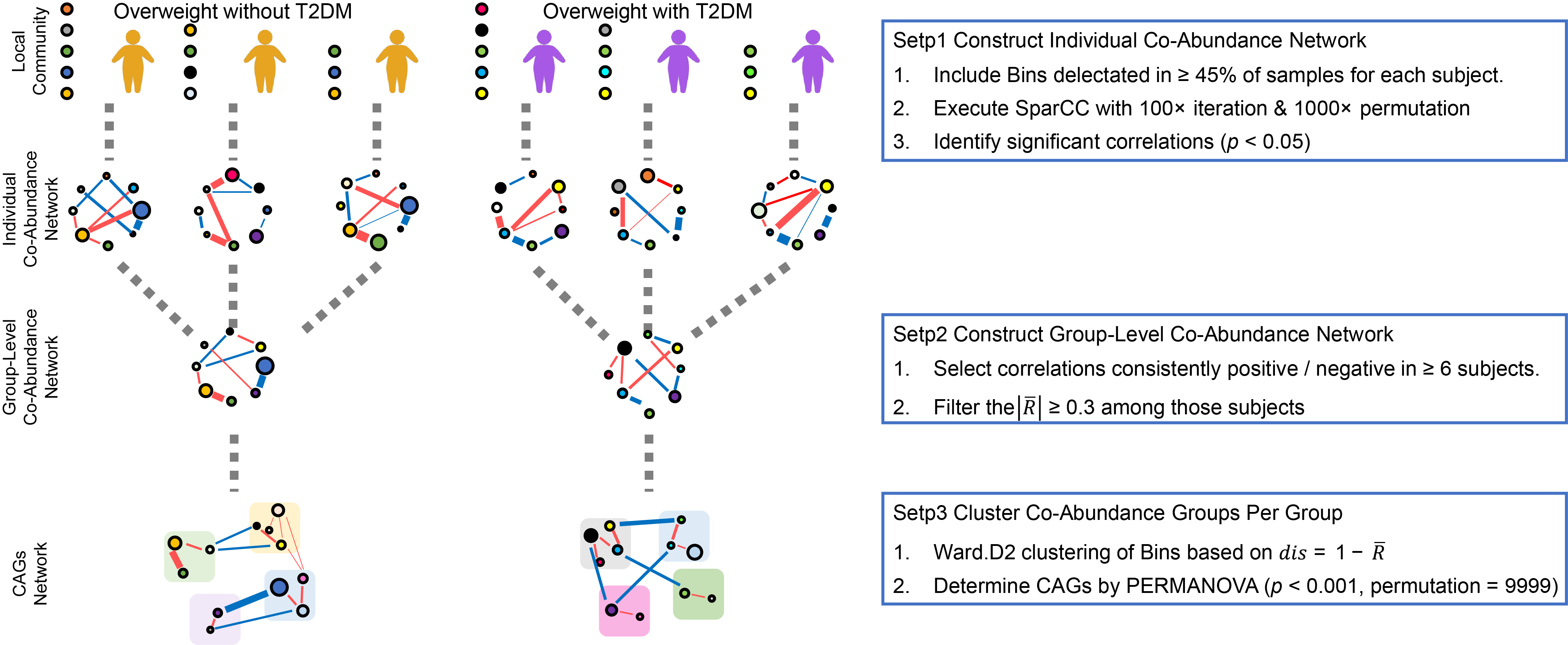
**Figure S4 Co-occurrence network construction method.** Individual-level co-abundance networks are constructed first (top), followed by derivation of group-level networks based on consensus across individuals (middle), and subsequent clustering into co-abundance groups (bottom).

#
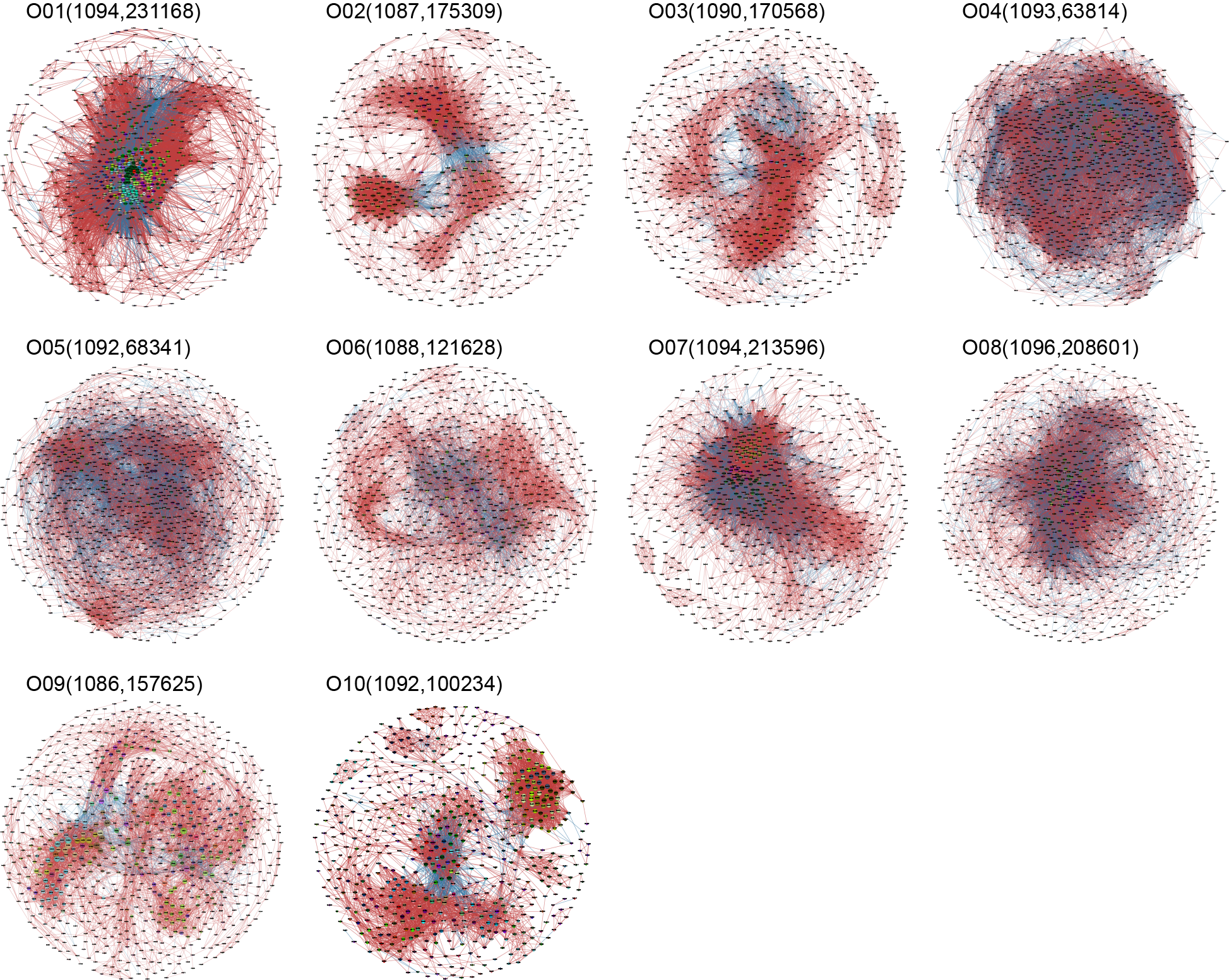
Figure S5 Co-abundance network in overweight participants. The number of nodes and edges in each subject’s network is indicated in parentheses.


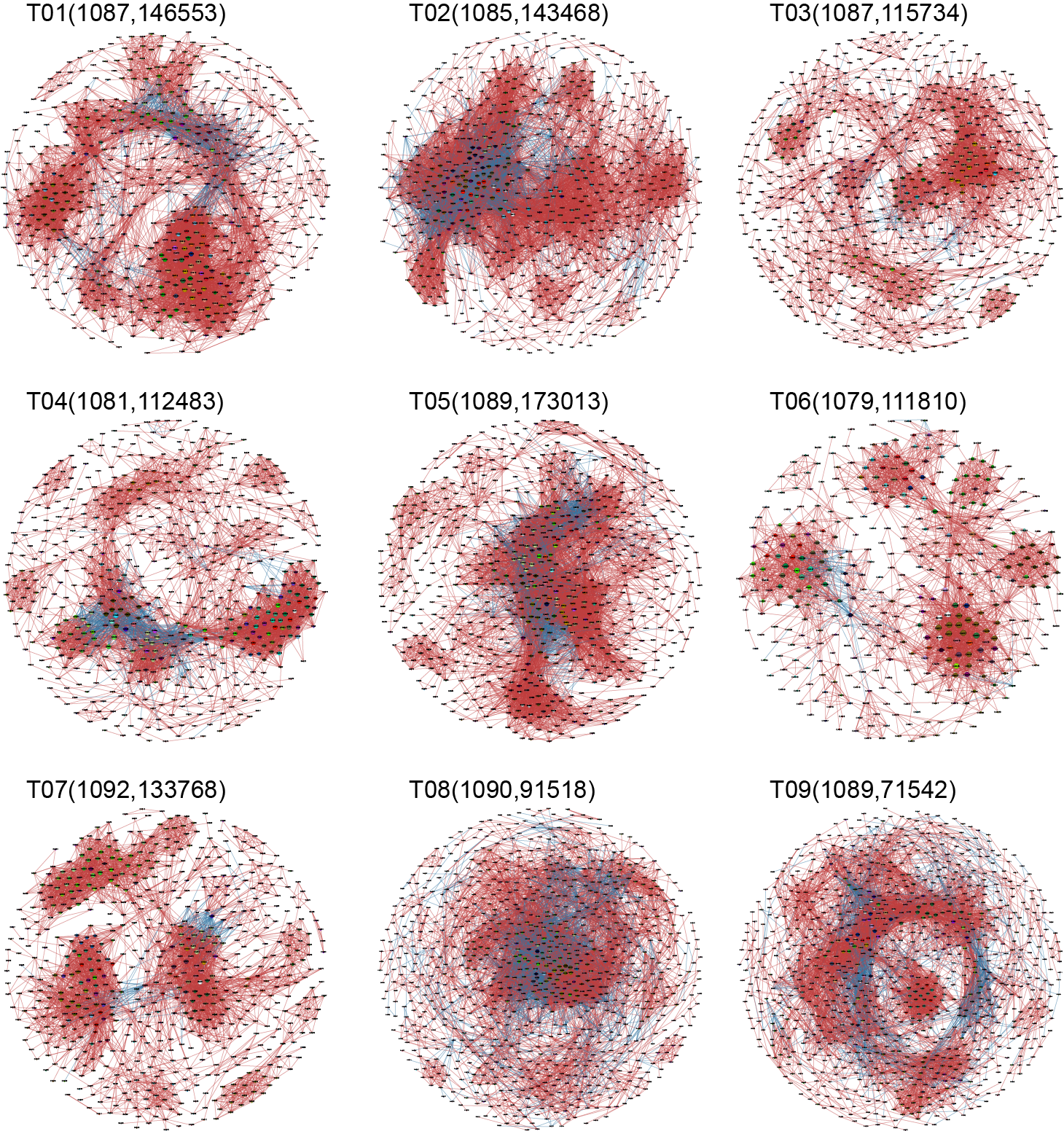


**Figure S6 Co-abundance network in overweight participants with T2DM.** The number of nodes and edges in each subject’s network is indicated in parentheses.


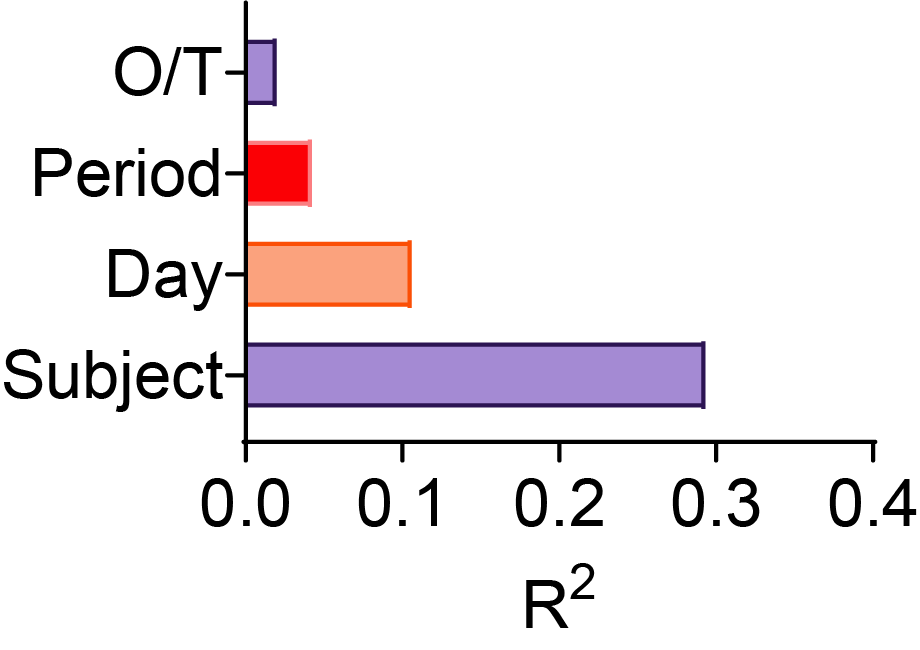


**Figure S7 Influence of participant factor on the fecal metabolite profile.** PERMANOVA test based on Euclidean distance with 999 permutations, BH-adjusted *p* < 0.05.


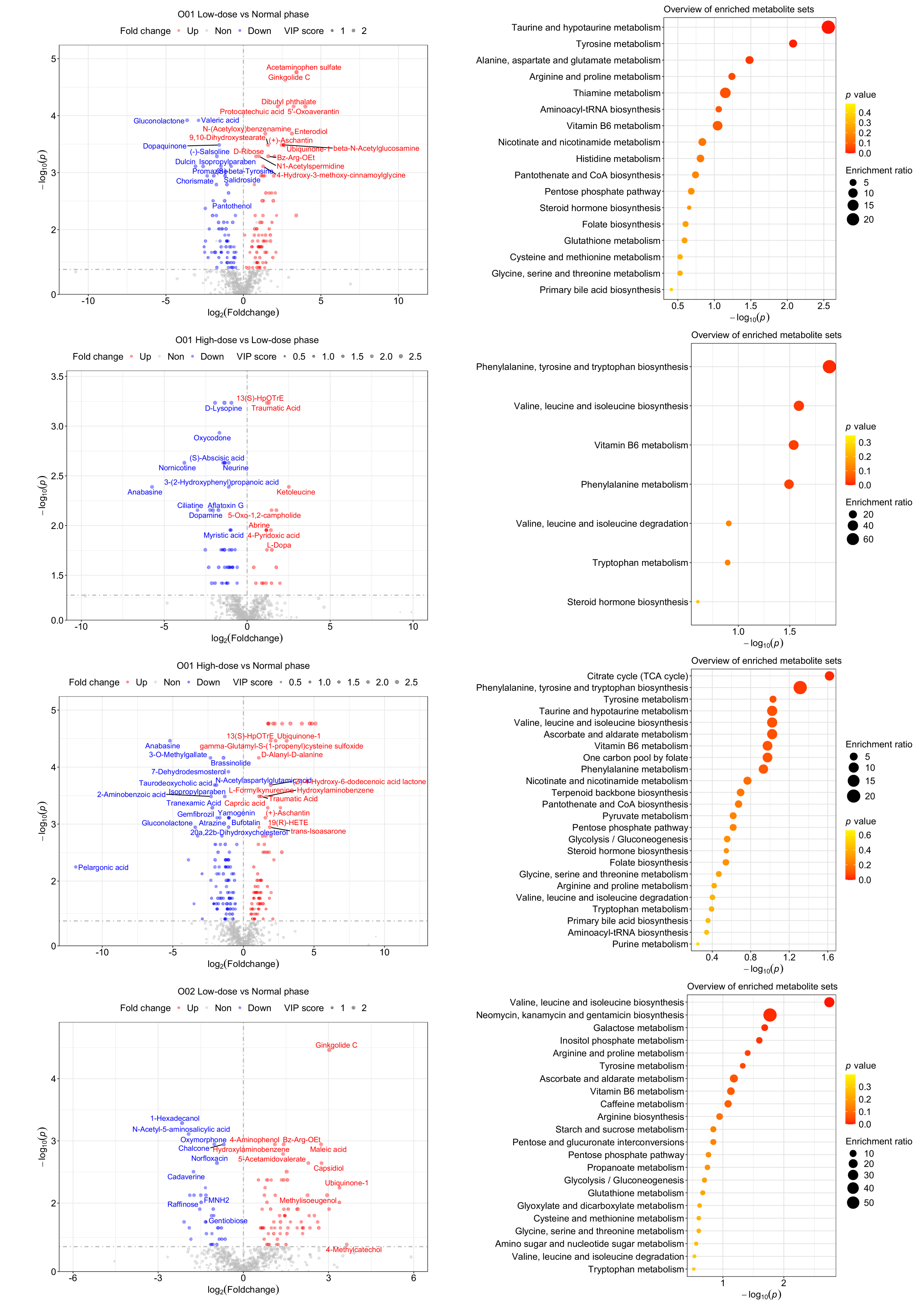

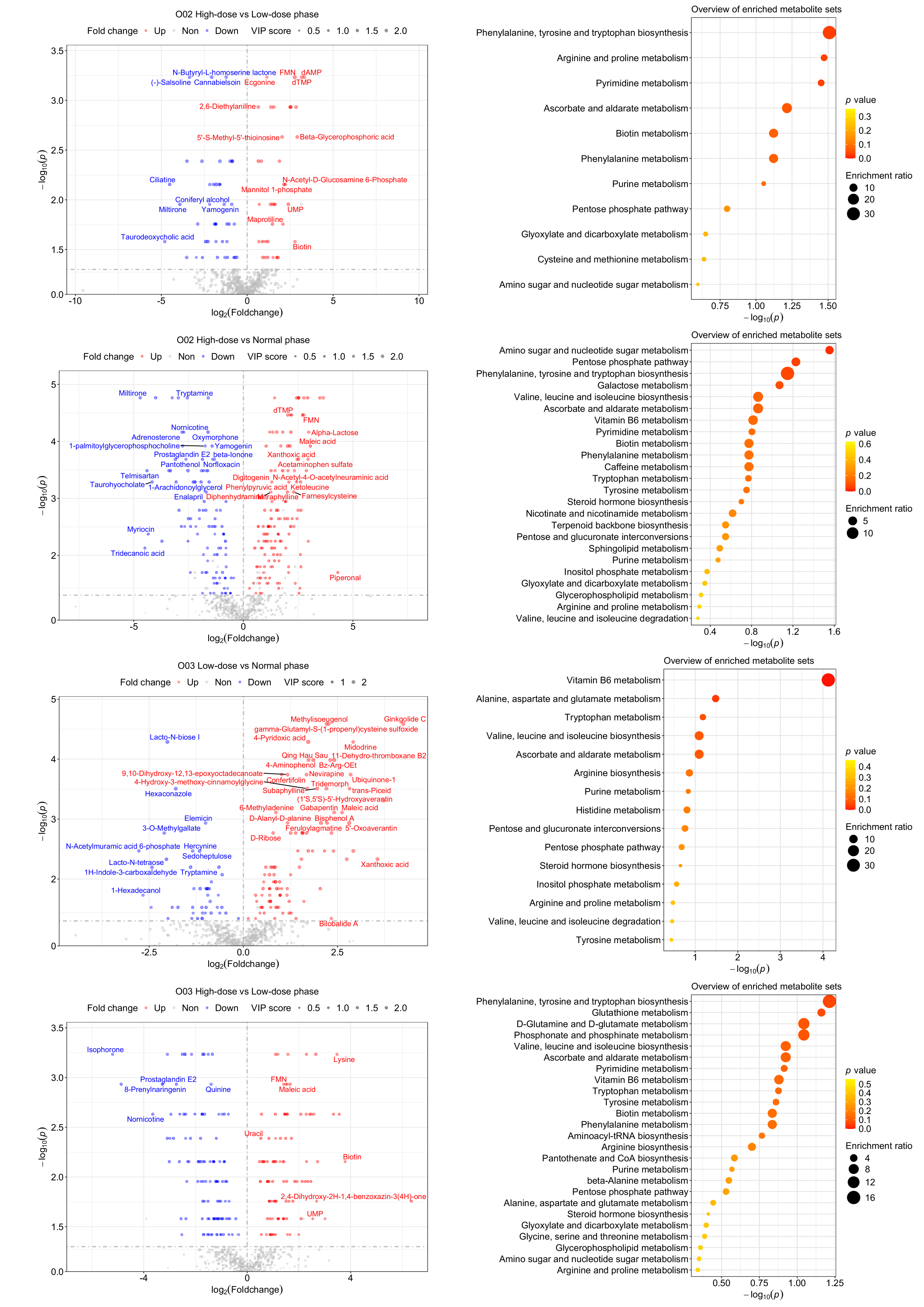

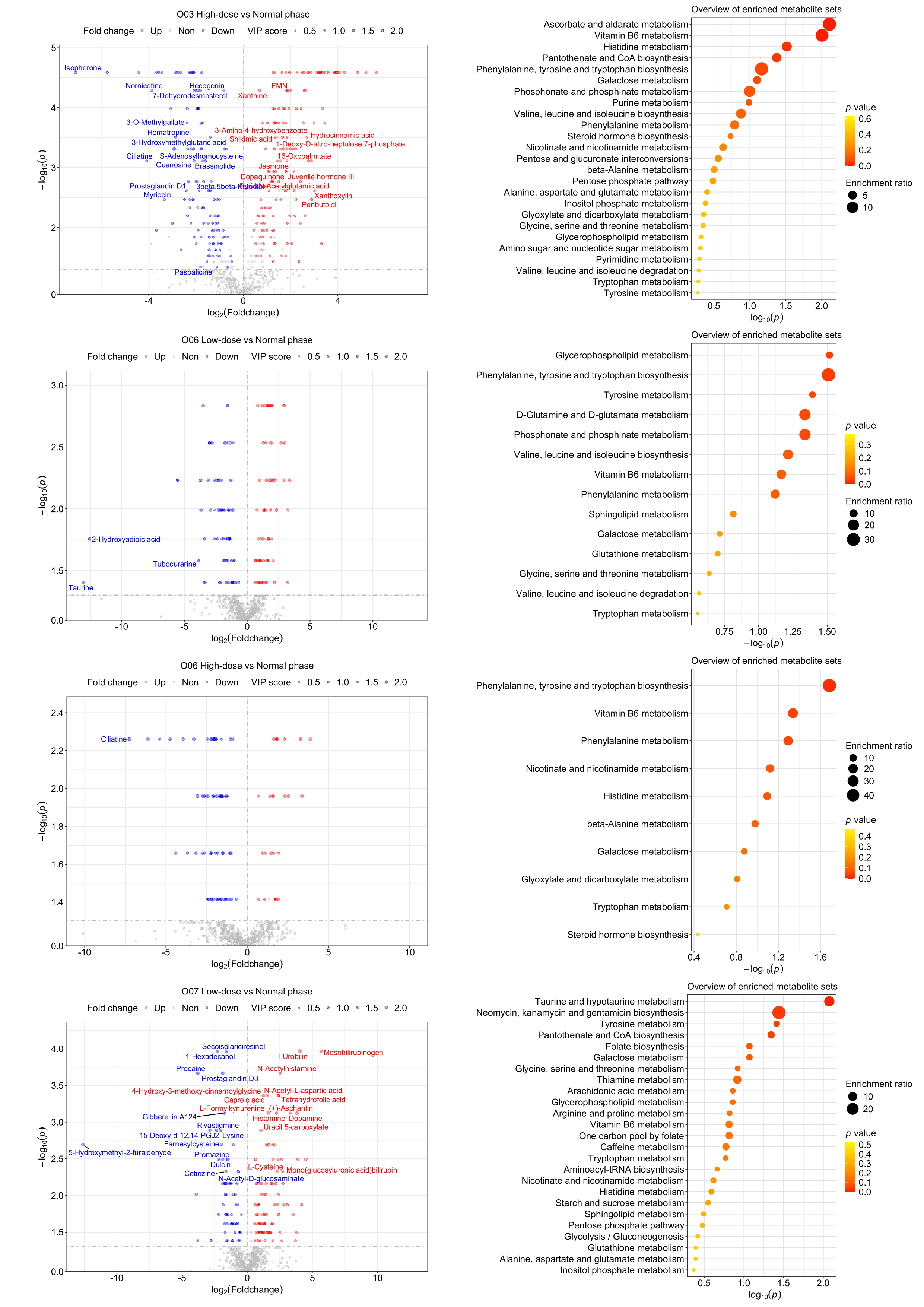

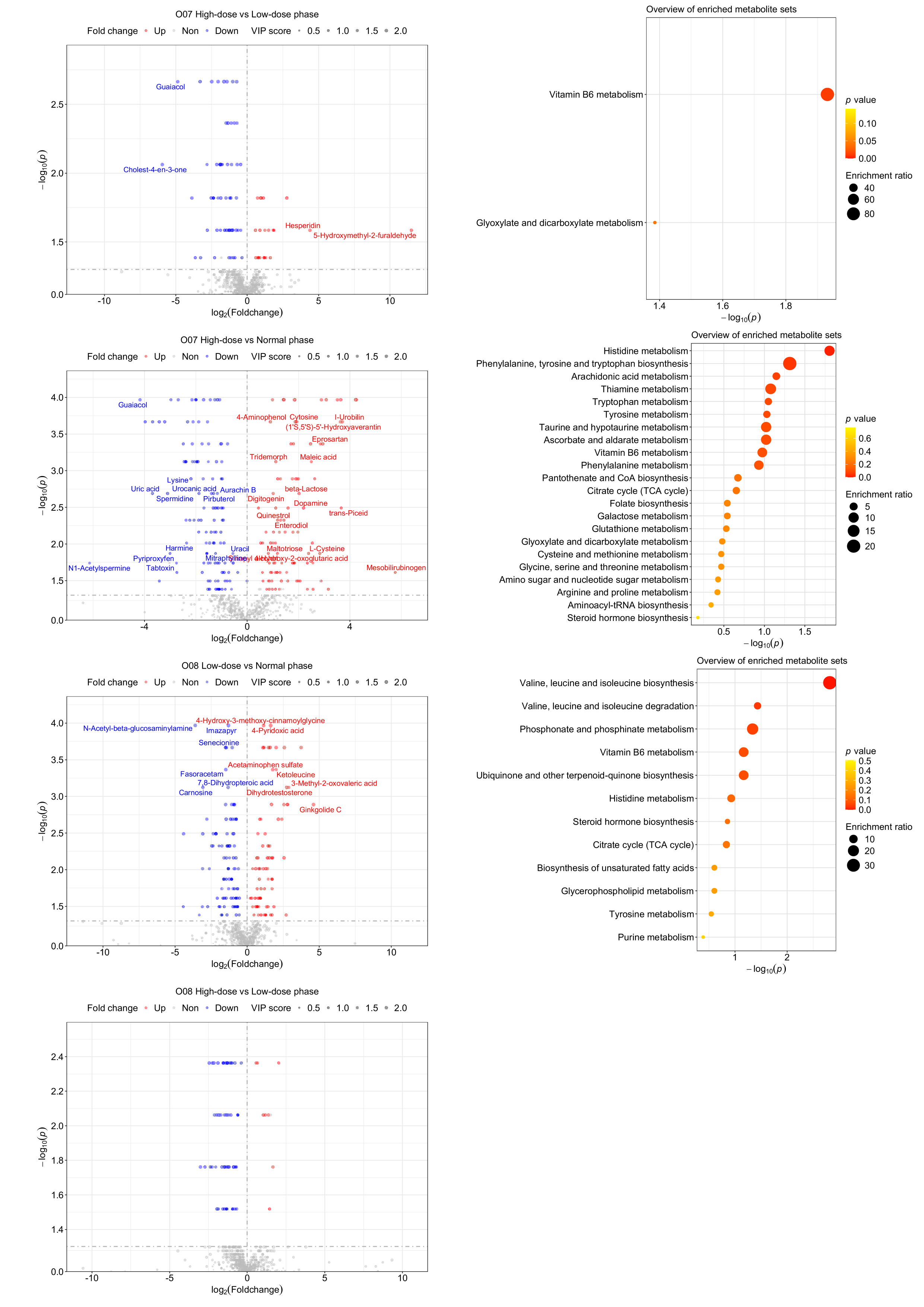

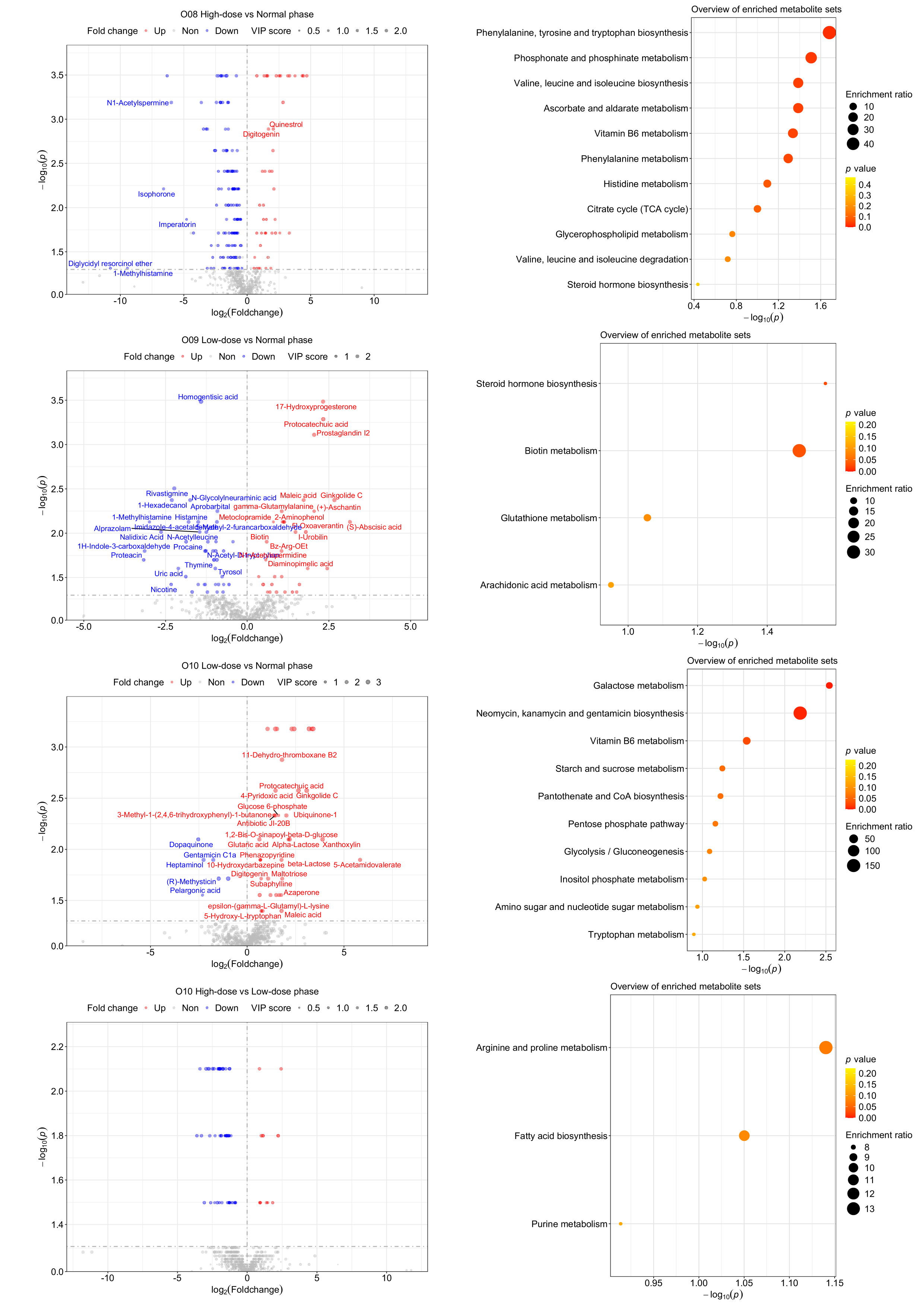

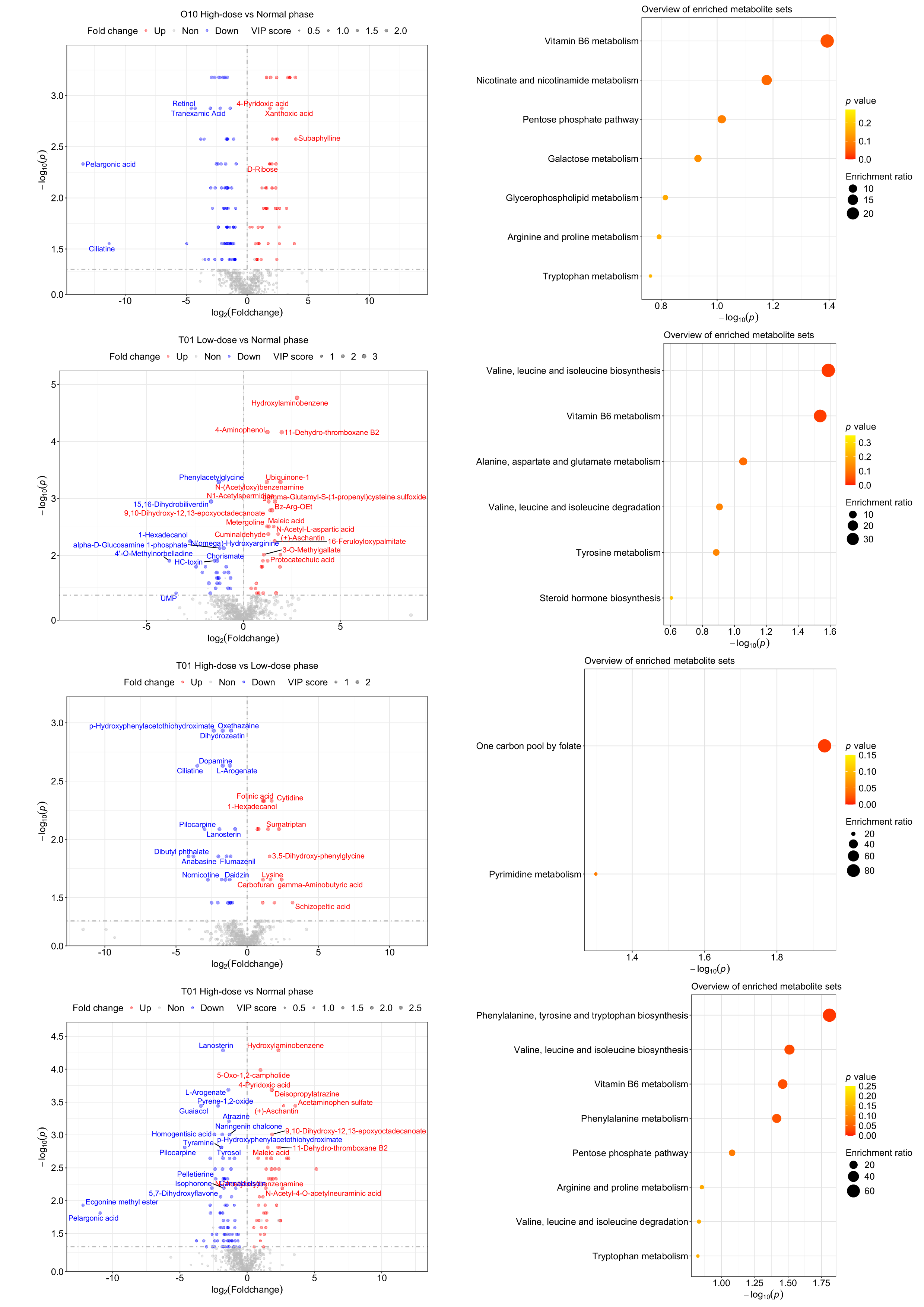

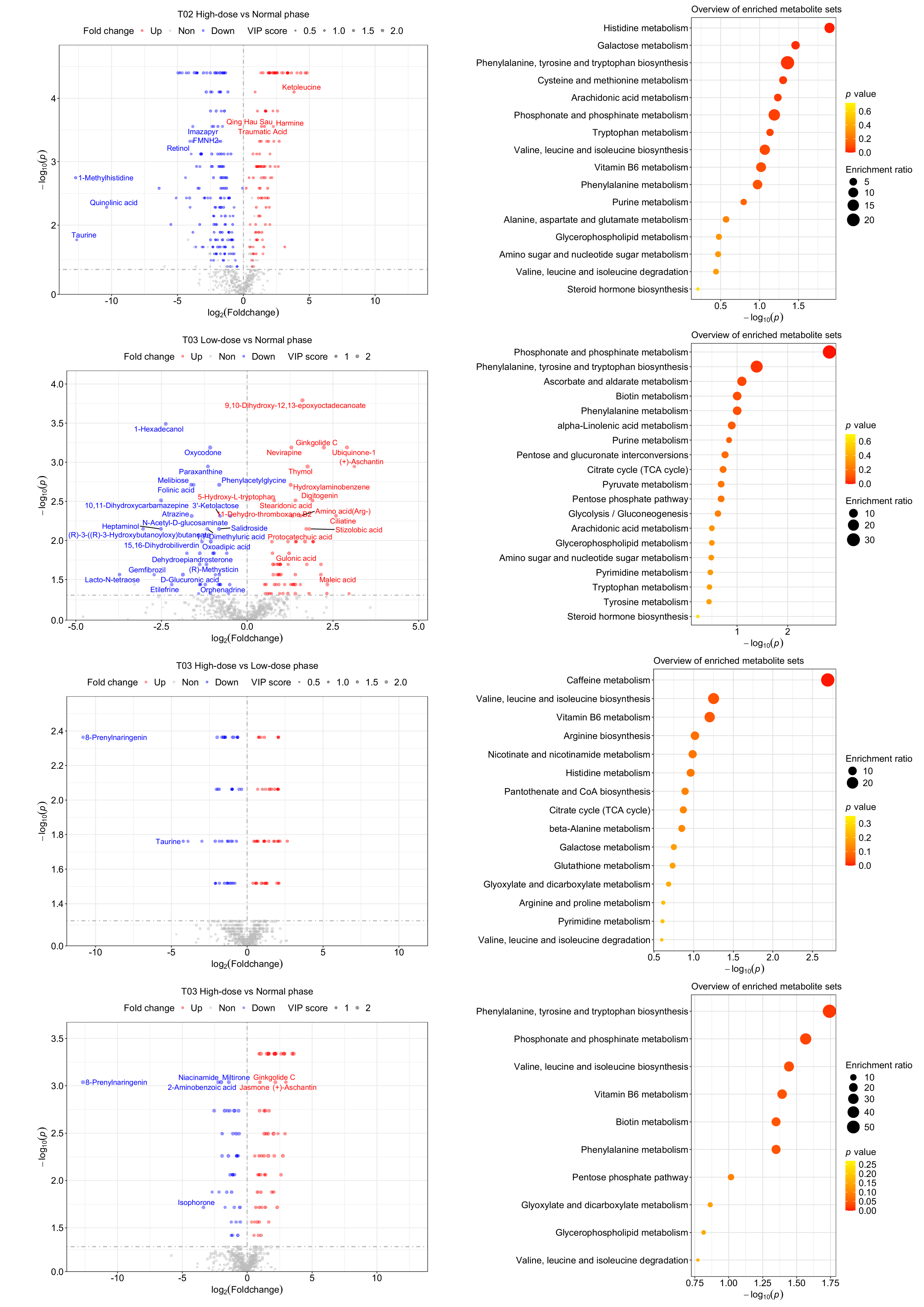

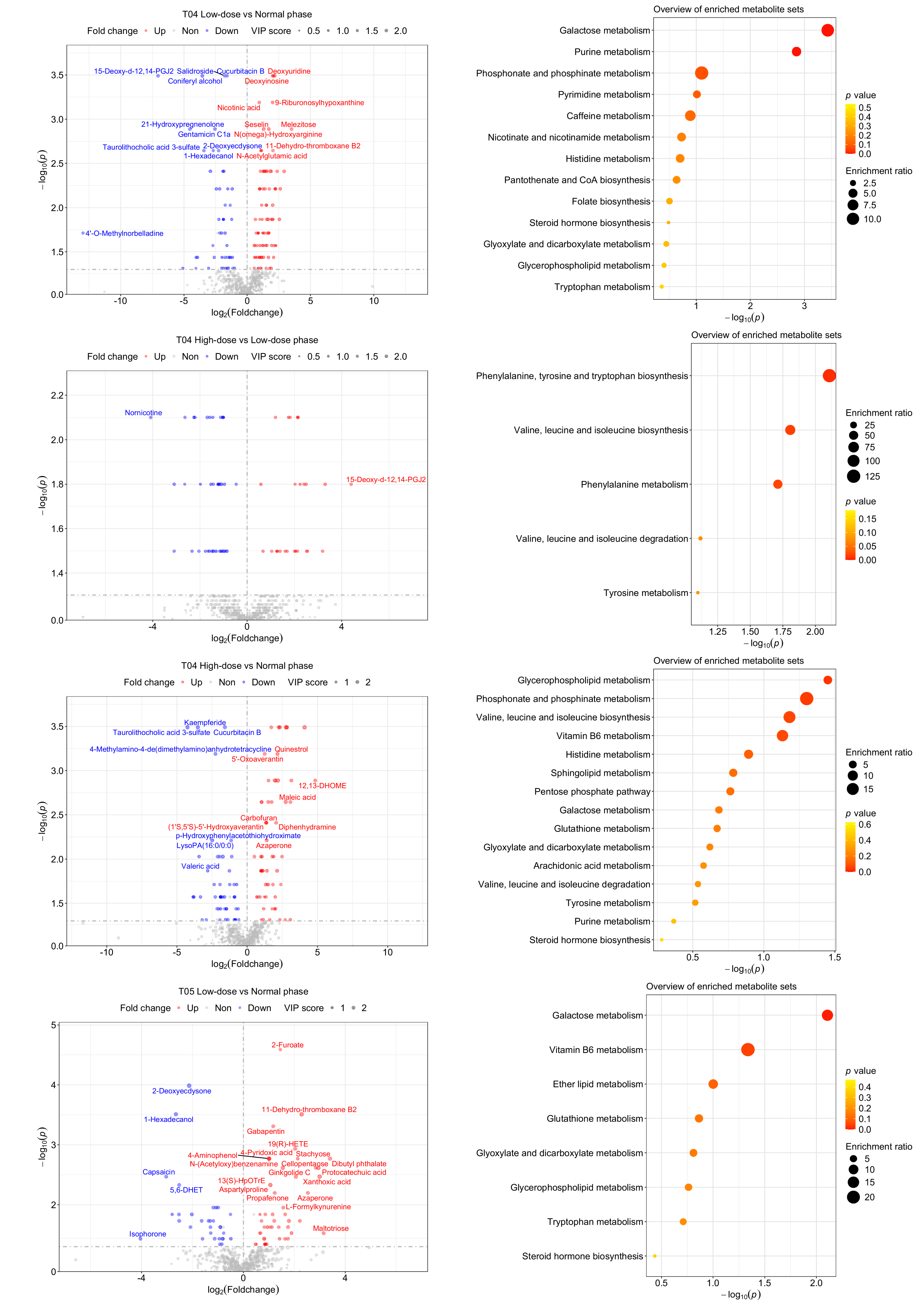

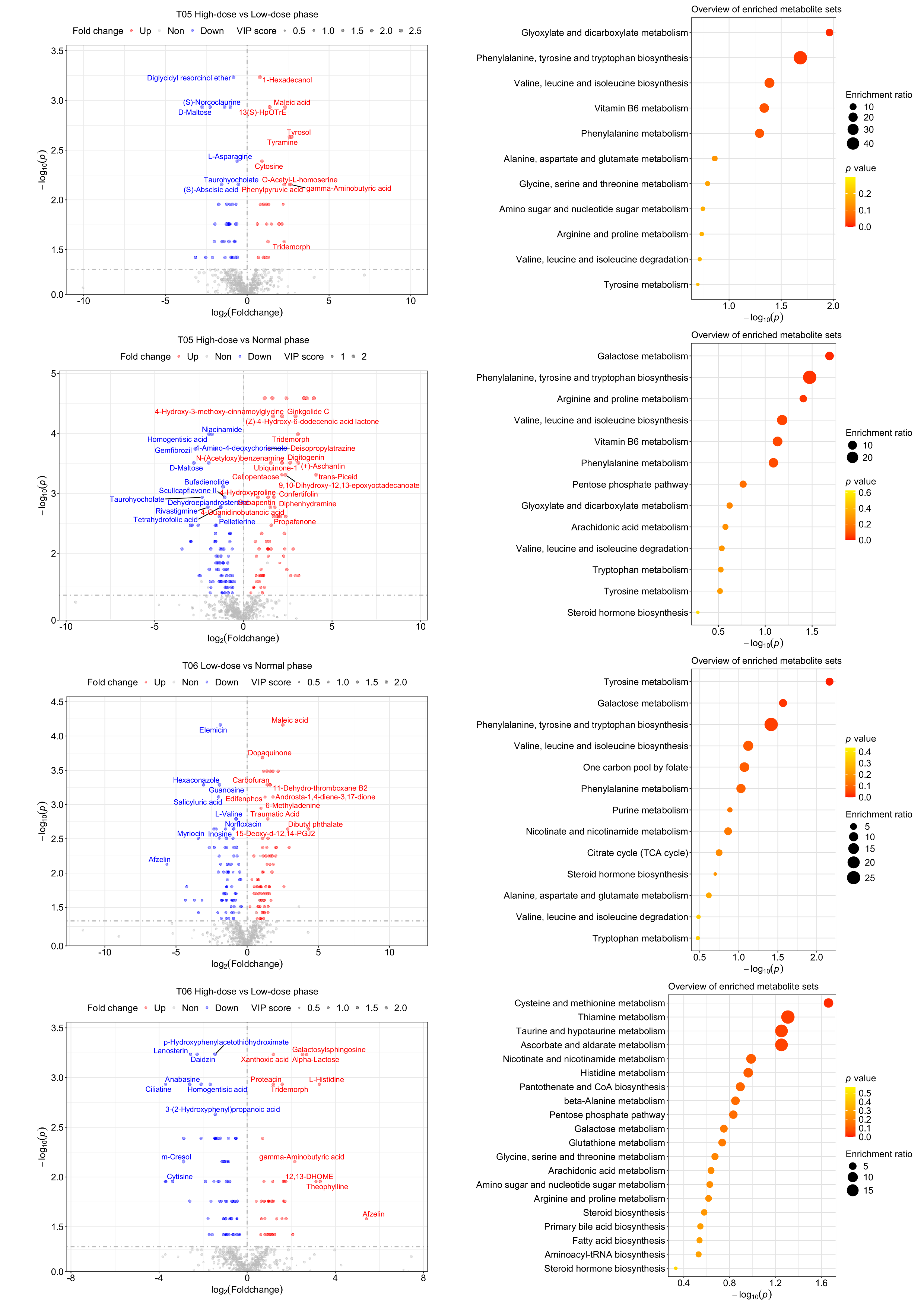

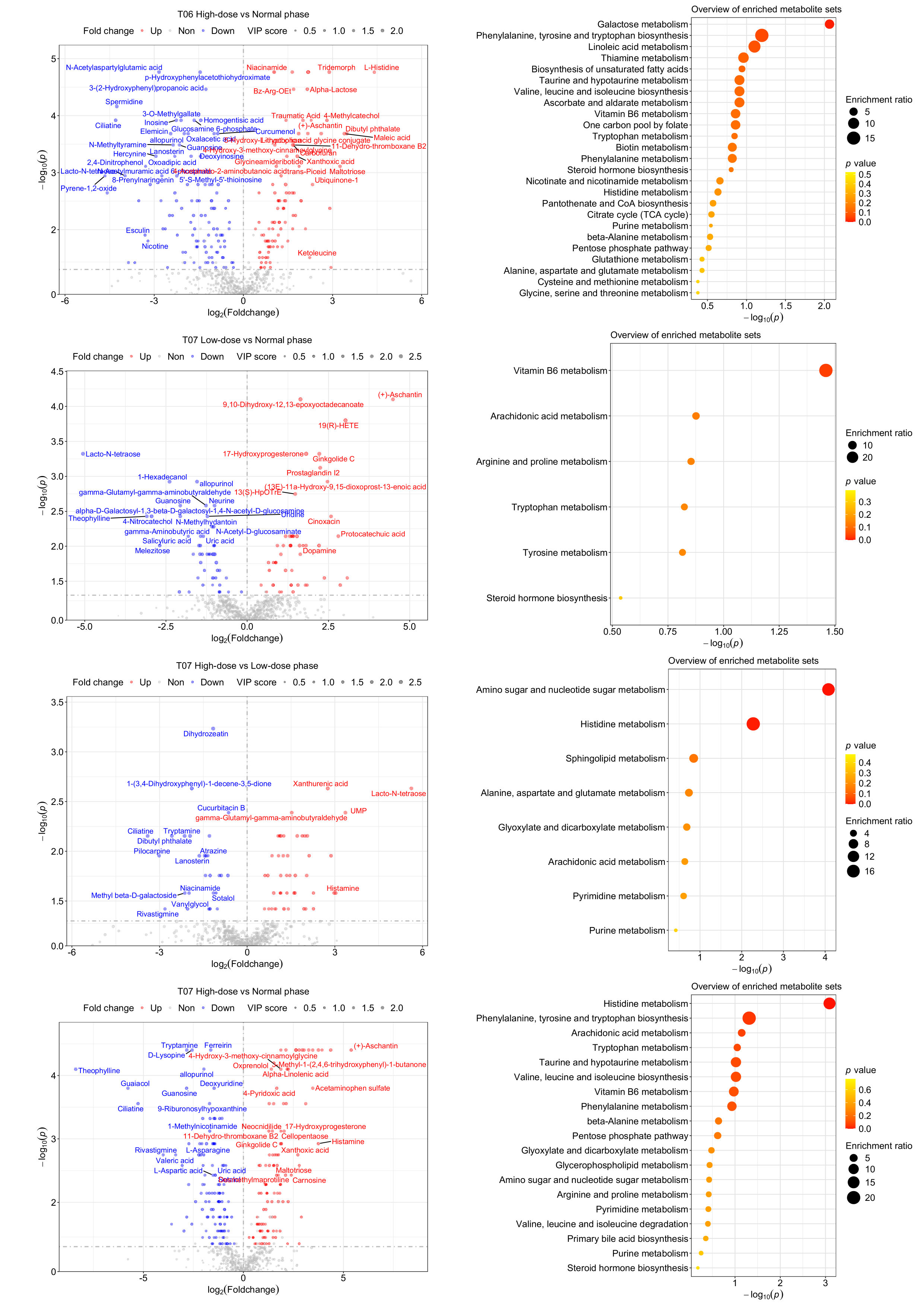

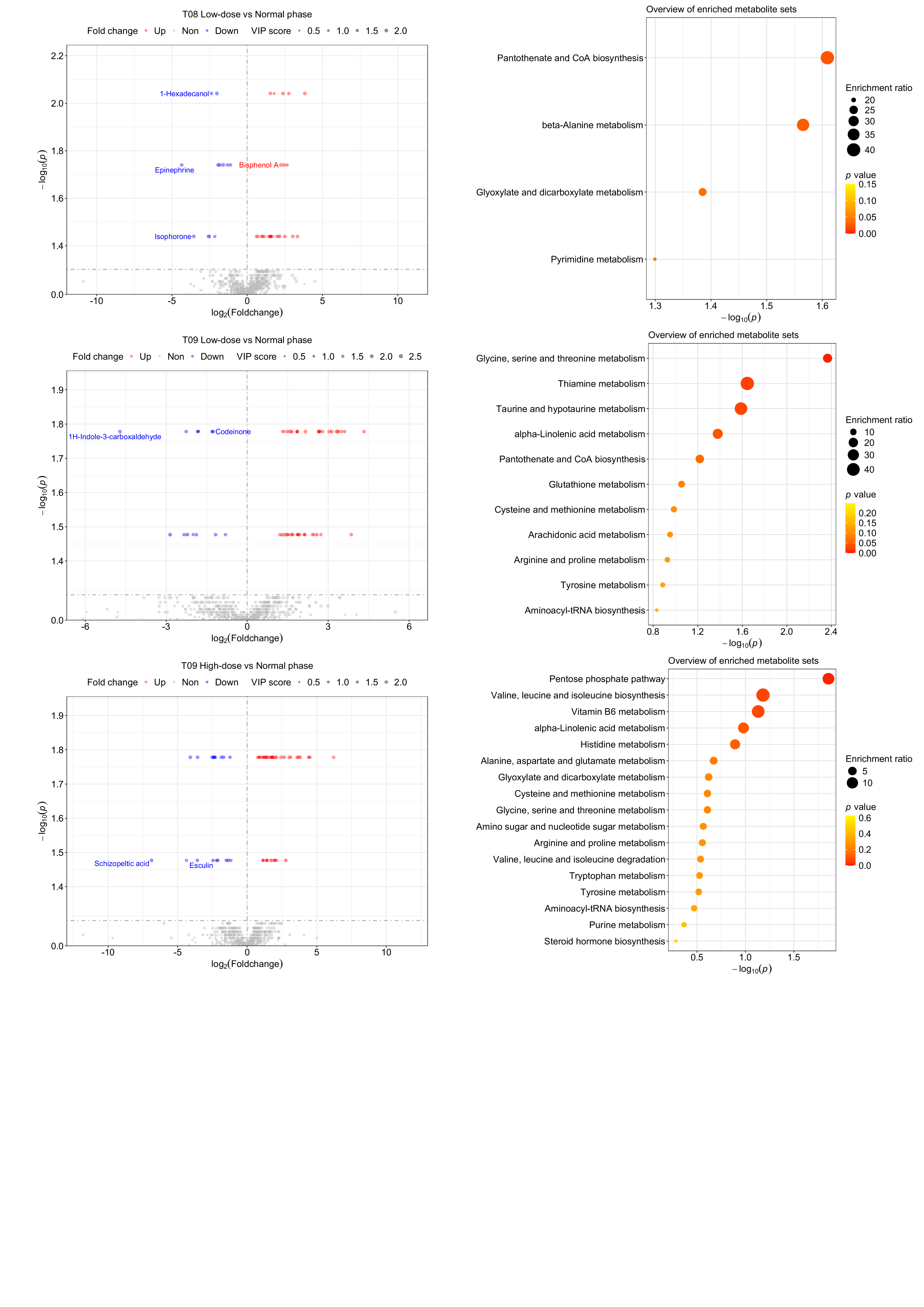
**Figure S8 Volcano plots of fecal metabolite changes and enriched pathways.** Exemplified by subject O01's comparison between normal diet and low-dose periods. Left: Volcano plot of metabolite changes. Each point represents a metabolite. Red points indicate metabolites that are upregulated during the low-dose phase, while blue points represent downregulated metabolites. Gray points indicate metabolites with no significant change. Point size corresponds to VIP value. Right: Enriched pathways based on upregulated metabolites after intervention. Point size indicates enrichment ratio and point color indicates statistical significance.

#
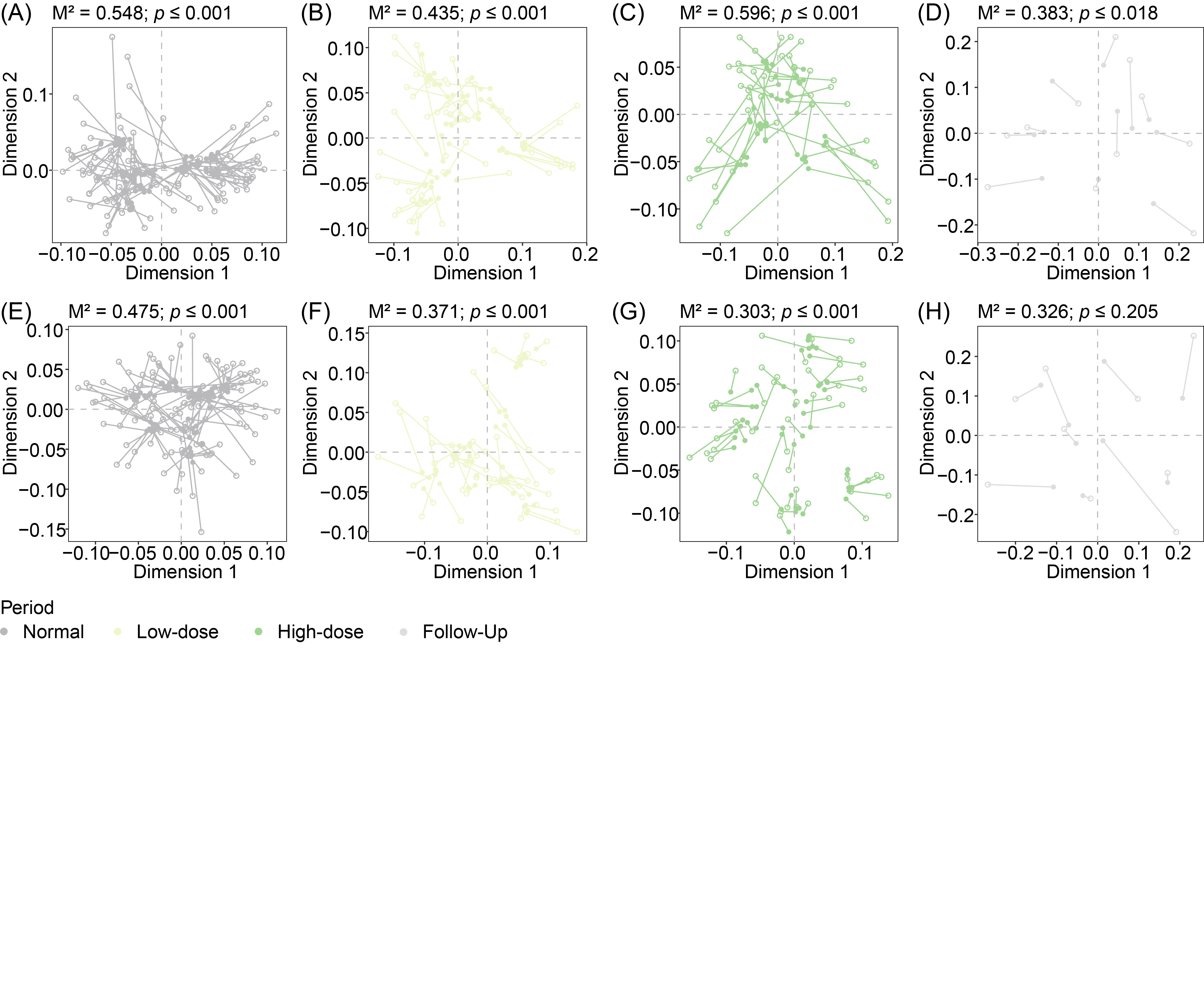
Figure S9 Similarity in the changes in gut microbiota structure and fecal metabolome characteristics during the trial. (A-D) Overweight group, (E-H) T2D group.


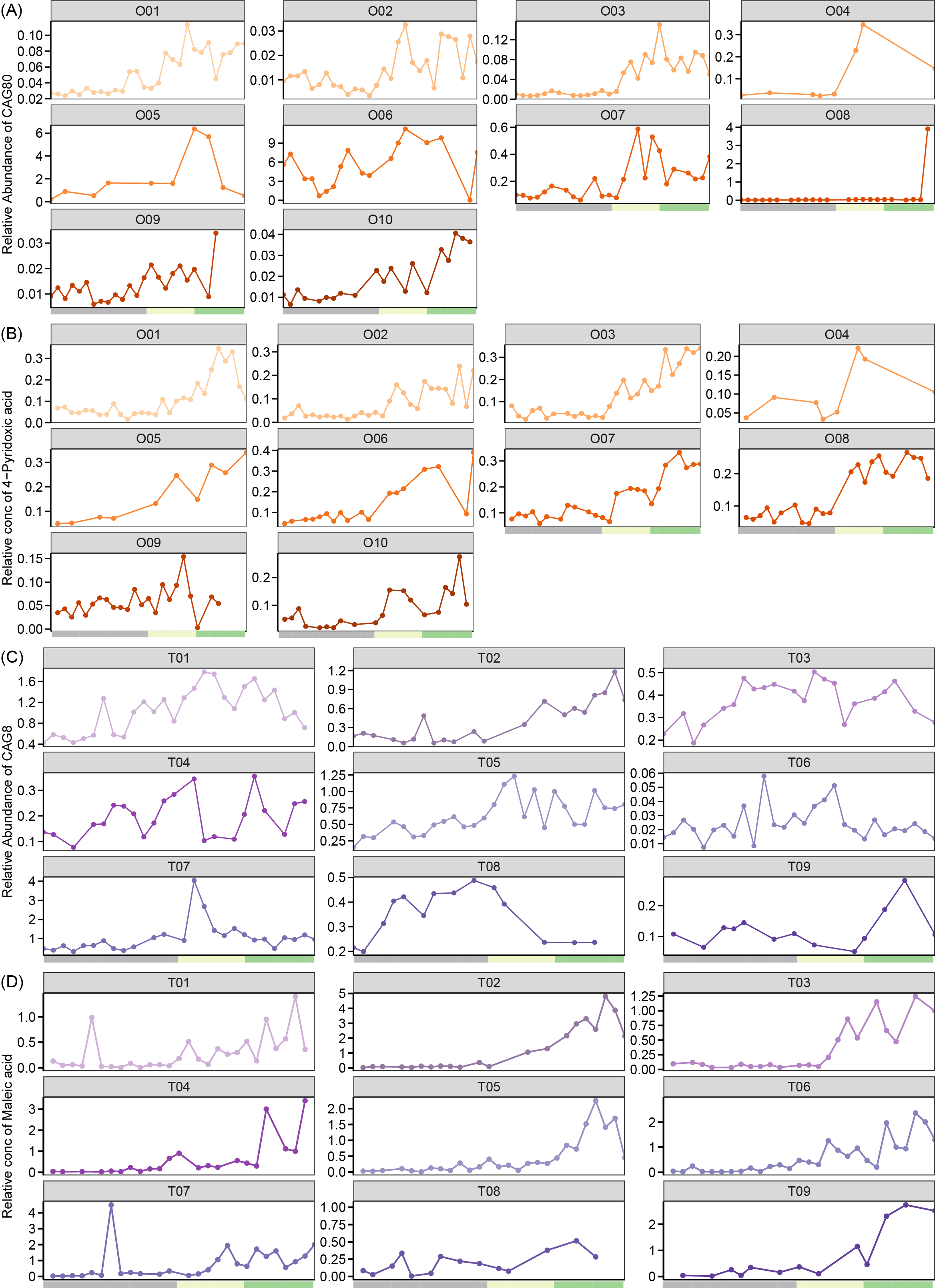
**Figure S10 Temporal changes in individual CAG and fecal metabolite levels during the trial.** (A and B) For the Overweight group: (A) CAG80 and (B) 4-phenylbutyric acid (4-PA). (C and D) For the T2D group: (C) CAG8 and (D) maleic acid.
